# Glioblastoma pseudoprogression and true progression reveal spatially variable transcriptional differences

**DOI:** 10.1186/s40478-023-01587-w

**Published:** 2023-12-04

**Authors:** Wesley Wang, Jonah Domingo Tugaoen, Paolo Fadda, Amanda Ewart Toland, Qin Ma, J. Brad Elder, Pierre Giglio, James Cancer Center Integrated Neuro-Oncology Team, Pierre Giglio, Shirley Ong, Clement Pillainayagam, Justin Gornanovich, Megan Gould, Judith Lima, Russell Lonser, Brad Elder, Douglas Hardesty, Timothy Lucas, Saman Ahmadian, Peter Kobalka, Diana Thomas, Wayne Slone, Arnab Chakravarti, Raju Raval, Sasha Beyer, Joshua D. Palmer, Dukagjin Blakaj, Erica Dawson, Erica Bell, José Javier Otero

**Affiliations:** 1 Department of Pathology, The Ohio State University Wexner Medical Center, The Ohio State University College of Medicine, 4166 Graves Hall, 333 W 10th Avenue, Columbus, OH 43210 USA; 2 Genomics Shared Resource-Comprehensive Cancer Center, The Ohio State University, Columbus, OH USA; 3 Department of Cancer Biology and Genetics, The Ohio State University, Columbus, OH USA; 4 Department of Biomedical Informatics, The Ohio State University, Columbus, OH USA; 5 Department of Neurosurgery, The Ohio State University Wexner Medical Center, Columbus, OH USA; 6 Department of Neuro-Oncology, The Ohio State University Wexner Medical Center, Columbus, OH USA; 7 The James Cancer Hospital and Solove Research Institute, Columbus, OH USA

**Keywords:** Glioblastoma, Pseudo-progression, Novel enhancement, Pathology informatics, Clinical decision-making

## Abstract

Post-resection radiologic monitoring to identify areas of new or progressive enhancement concerning for cancer recurrence is critical during patients with glioblastoma follow-up. However, treatment-related pseudoprogression presents with similar imaging features but requires different clinical management. While pathologic diagnosis is the gold standard to differentiate true progression and pseudoprogression, the lack of objective clinical standards and admixed histologic presentation creates the needs to (1) validate the accuracy of current approaches and (2) characterize differences between these entities to objectively differentiate true disease. We demonstrated using an online RNAseq repository of recurrent glioblastoma samples that cancer-immune cell activity levels correlate with heterogenous clinical outcomes in patients. Furthermore, nCounter RNA expression analysis of 48 clinical samples taken from second neurosurgical resection supports that pseudoprogression gene expression pathways are dominated with immune activation, whereas progression is predominated with cell cycle activity. Automated image processing and spatial expression analysis however highlight a failure to apply these broad expressional differences in a subset of cases with clinically challenging admixed histology. Encouragingly, applying unsupervised clustering approaches over our segmented histologic images provides novel understanding of morphologically derived differences between progression and pseudoprogression. Spatially derived data further highlighted polarization of myeloid populations that may underscore the tumorgenicity of novel lesions. These findings not only help provide further clarity of potential targets for pathologists to better assist stratification of progression and pseudoprogression, but also highlight the evolution of tumor-immune microenvironment changes which promote tumor recurrence.

**Supplementary Information:**

The online version contains supplementary material available at 10.1186/s40478-023-01587-w.

## Introduction

Glioblastomas (GBs) are amongst the most common of primary brain malignancies comprising 14.3% of all CNS tumors and 49.1% of malignant tumors [1]. Current standard of care for this condition includes a maximal-safe surgical resection of the lesion followed by cycles of radiation and chemotherapy (chemoRT) [2]. The cancer’s inevitable recurrence following chemoRT however requires serial MRI evaluation to identify novel contrast enhancing lesions indicating potential recurrence. Increased contrast agent uptake immediately or soon after chemoRT which eventually dissipates however complicates disease interpretation as these treatment related changes mimic the appearance of true cancer progression (PD) [3]. Termed pseudoprogression (psPD), psPD requires drastically different treatment to that of PD. While true cancer progression necessitates the need for treatment revision or palliation, patients experiencing psPD events can be carefully monitored or symptomatically treated with steroids without changing their oncologic management [3]. Accurate stratification of psPD from PD is critical to prevent delayed treatment in progressive patients or unnecessary therapy changes in pseudoprogressive patients. Furthermore, in patients enrolled in clinical trials for GB management, presence of novel enhancement may freeze study participation until a lesion is properly diagnosed [4].

Currently, while conventional multiparametric MRI may capture the initial enhancement, accurately stratifying an event as PD or psPD is not possible at initial presentation [5]. Clinicians often choose to closely monitor a patient short-term to see whether a lesion dissipates radiologically or choose to more aggressively sample the site surgically to determine whether a lesion appears more progressive or pseudoprogressive histologically. However, no standardized clinical guideline exists to effectively differentiate these two entities based upon histology. Many sites have used a combination of markers to evaluate cellular proliferation concerning for cancer recurrence against areas of necrosis and inflammation which may better represent treatment-related damages. Even still, past work has suggested that a large portion of biopsied patients present with mixed histology that highlight areas of tumor and treatment effect [6]. In summary, neuropathologists are challenged with characterizing areas of hypercellularity and immune infiltration which neuro-oncologists utilize in deciding whether to revise a patient’s oncologic management or continue conservative monitoring.

In the present study, we assessed the validity of using proliferative stem cell and immune signatures as markers of disease outcome by clustering an online RNAseq repository of recurrent GB samples using an 83-gene signature representing these processes. Additionally, second surgery lesion samples were retrospectively collected to explore the efficacy of our disease management through patient survival and to characterize the molecular landscape of these lesions. Due to added complexity from lesion heterogeneity however, admixed PD/psPD samples were used to generate spatially derived imaging and expression data to explore novel differences in the tumor microenvironment not applied to current clinical workflows.

## Methods

### Patient data collection

Patients seen at The Ohio State University for GB management from January 2012 to March 2020 were evaluated retrospectively for history of novel enhancement following ChemoRT. Study execution was performed under IRB study number 2020C0062. Patients were recorded in REDcap based upon the following inclusion criteria: (i) GB management by Ohio State and (ii) *MGMT* methylation testing; and exclusion criteria: (i) *IDH*-mutation, (ii) history of low-grade glioma, and (iii) patients with poor documentation of disease course [7, 8].

Patients subsequently defined as having psPD in the study was based on the following definitions: (i) detection of new or progressive enhancement within 6 months of ChemoRT, (ii) clinico-pathologically confirmed treatment reaction, and (iii) no prior history of progression. Patients defined as having PD in the study was based on the following definitions: (i) clinico-pathologically confirmed cancer recurrence and (ii) no prior history of progression. Definition for inclusion time points for psPD are based upon 6 month recommended surveillance times [9, 10].

### Online RNAseq analysis

Recurrent GB bulk RNA sequencing (RNA-seq) analysis was performed using publicly available data from the Chinese Glioma Genome Atlas (CGGA) from dataset mRNAseq_693 [11]. Normalized data [n = 109 samples] was clustered using select immune and cancer cell signature markers as described in Additional file 1: Table S1 using RStudio and Rtsne [12–20]. Cluster determination was performed using kMeans and k was selected using the silhouette method [21, 22]. Furthermore, validation of clustering significance was performed by randomized permutation in R. Dummy variables were generated by randomly scrambling our initial dataset used to generate clusters. Subsequently, the average within cluster distance of points within a grouping and average between cluster distance of points amongst other groups were measured by randomly sampling cases and repeating permutations 10,000 times for both our actual and randomly scrambled data.

Accompanying survival data from the CGGA was utilized and visualized using R survival and survminer packages [23, 24]. Immune enrichment was calculated using xCell and cell populations showing zero variance across groups were removed for visualization [25]. Weighted gene correlation network analysis (WGCNA) was performed using the R WGCNA package and modules with non-significant enrichments (*p* < 0.05) of biologic processes and molecular functions based on gene ontology (GO) were removed in visualization [26].

### Bulk Ncounter analysis

Tissue specimens used for subsequent molecular analysis were selected based upon clinical history as either PD or psPD using the above listed clinical definitions and final diagnosis rendered by an interdisciplinary tumor board. Neuropathologic assessment was based upon cellular proliferation showing atypical nuclear morphologies through immunohistochemical evaluation of Ki67, Olig2, and p53 for cancer recurrence. Treatment reaction was identified by quiescent cellularity with active immune response based on CD68, CD163, or CD45. All samples were surgically collected from original second surgery and stored as archival formalin-fixed paraffin embedded (FFPE) tissue. Specifically, 60 samples [PD: 31; psPD: 29] were selected that fell within study defined timepoints and had adequate tissue for molecular study. Samples were then selected for RNA extraction using a Norgen FFPE RNA Purification Kit [Cat. 25300, 25400] following the manufacturer's protocol. Quality control validation for RNA concentration, purity, and degradation was performed using a ThermoFisher NanoDrop, ThermoFisher Qubit, and Agilent Tapestation. In total, 48 samples [PD: 27; psPD: 21] passed quality control and were used for nCounter analysis (Additional file 1: Fig. S1).

Bulk expression analysis of second surgery samples from OSU was performed using a NanoString nCounter Pan-cancer 360 panel [Cat. PSTD-H-T360-12]. Data normalization and differential expression analysis were performed using NanoString Rosalind Platform [27, 28]. Survival visualization of clinical samples from bulk analysis was performed using R survival and survminer packages and defined as time from second surgery [23, 24]. Differential expression visualization was performed using EnhancedVolcano and gene set enrichment was assessed using GO-analysis [29–31]. Differentially expressed genes were further assessed using QIAGEN Ingenuity Pathway Analysis (IPA) to generate summary networks correlating potential driver genes to pathway enrichment [QIAGEN Inc., https://digitalinsights.qiagen.com/IPA] [32]. Sample clustering and validation were performed as described in the online RNAseq methods subsection using top 150 differentially expressed genes (Additional file 1: Fig. S2).

### Automated image segmentation

Clinical imaging of sample pathology was collected from Phillips Image Management System. Representative images were collected from 8 clinical samples stained and scanned for H&E, CD163, Olig2, Ki67, and p53. In total, 260 representative images at approximately 4000 × 2000 pixels were taken for each stain with accompanying negative/positive control images when available. Scanning of slides was performed using a Phillips Ultra Fast Scanner. Image processing schematics can be found in Additional file 1: Fig. S3. H&E scanned images were deconvoluted to separate hematoxylin and eosin stain, grayscaled, and segmented using an Otsu thresholding approach [33]. Olig2, Ki67, and p53 were alternatively deconvoluted to separate hematoxylin and 3,3'-diaminobenzidine (DAB) stain, grayscaled, and Otsu thresholded. Deconvolution was performed using python Scikit-image imported into R studio using reticulate [34, 35]. While image grayscaling and thresholding were processed using EBimage in R [36]. In contrast, CD163 was segmented using a UNET convolutional neural network (CNN) in python using tensorflow due to high DAB background staining [37–39].

Model development was performed in python using 6,524 image tiles at 512 × 512 pixels using a UNET architecture with default hyperparameters and DAB noise augmentation [37]. Ground truth image masks to assess model performance were manually segmented by a trained observer and separated to segment cellular bodies and processes to develop two parallel CNN models (Additional file 1: Fig. S3). Two hundred and sixty representative images were taken from relevant regions of interest and split into 512 × 512 pixel tiles composing a total dataset of 5,809 CD163 image tiles. All tiles were passed by both CNN models to generate prediction maps for cellular bodies and processes, global thresholded, and merged to generate whole cell image masks for use in R.

### Image feature analysis

Image segmentation masks were overlayed onto original images to calculate cell shape, cell moment, pixel intensity, and pixel texture features for each segmented cell using EBimage [36]. Low intensity staining and small object artifacts [objects with punctate segmentation non-representative of true cells] were filtered (Additional file 1: Fig. S3). Low intensity staining was filtered using a random forest-based classifier to classify objects as either positive or negative for true signal [40]. Negatively labeled cells were subsequently filtered. Small object filtration was performed by removing objects showing zero area from feature extraction. Retained segmentations were utilized to calculate cell spatial distribution, pixel DAB to hematoxylin ratio, and cell morphology clustering [41].

Cell spatial distribution was calculated by extracting object x and y image positions from cell moment features in H&E images to calculate the nearest neighbor distance of a cell from its hundredth nearest neighbor in each image. Pixel DAB to hematoxylin ratio was calculated in Olig2, Ki67, p53, and CD163 images by extracting segmentations for DAB and hematoxylin layers and divided as the ratio of DAB^+^ pixels to hematoxylin^+^ pixels in an image. Cell morphology clustering was calculated by extracting cell shape and moment features (aside from x and y positions) for each cell segmentation and mapping cells using uniform manifold approximation and projection (UMAP) dimensionality reduction [42]. Eight thousand cells were randomly selected per group (16,000 total) for each stain to both control for uneven distribution of cell segmentations from PD and psPD events and reduce computational complexity. Identification of cell clusters in UMAPs was performed using DBscan in R and requiring clusters to at minimum be composed by 160 cells in a cluster [43].

### GeoMx digital spatial profile analysis

Spatially derived transcriptome analysis was performed using a NanoString GeoMx Digital Spatial Profiler using NanoString Human Whole Transcriptome Atlas probes. Tissue samples, clinical data, and definitions were collected as delineated above. Eight samples [PD: 4; psPD: 4] were sectioned at 5 microns after being evaluated by a neuropathologist for evidence of tissue heterogeneity (Additional file 1: Table S2; Additional file 1: Fig. S1). Ground truth clinical outcome was established retrospectively based on neuro-oncology clinical reports of patient disease progression. Sections were stained with Novus Sox10-Alexa Fluor 647 [Cat. NBP2-59621AF647], Santa Cruz CD68-Alexa Fluor 594 [Cat. sc-20060 AF594], and NanoString Syto13 [Cat. S7575] conjugated antibodies for digital spatial profiling (DSP) analysis. Exposure rates for the mentioned antibodies on the GeoMx platform were set at 300 ms for Sox10, 250 ms for CD68, and 200 ms for Syto13. Preparation of slides were done following the manufacturer's protocol. Regions of interest (RoIs) selection was performed using fluorescent staining and overlayed with digital pathology IHC for H&E, CD163, Olig2, Ki67, and p53 to select relevant regions based on the following histology definitions: Normal histology [predominated by normal neuron histology under H&E, normal surveillant microglia by CD163, normal oligodendrocyte growth following axons by Olig2]; Hypercellular histology [predominated by abnormal cellularity with evidence of active proliferation by Olig2, Ki67, and p53]; Inflammatory histology [predominated by increased macrophage/microglia infiltration by CD163]. All slides were reviewed by a neuropathologist and 192 total RoIs were collected. Normalization, differential expression analysis, and immune deconvolution of genes were performed using NanoString GeoMx software following the manufacturer's protocol [44–47]. Results were further visualized using Rstudio and gene cluster to clinical phenotype correlation was performed using the R WGCNA package [26].

Estimated expression of genes which did not pass quality control measurements were based upon machine learning imputation using CGGA RNAseq values. Genes with non-zero expression were calculated for sample expression variance in R within both CGGA and DSP datasets. Housekeeping genes were selected based on presence in both datasets and lower 20th percentile expression variance. Both datasets were normalized by dividing all genes by the identified lowest variance housekeeping gene. Normalized CGGA data was then passed through Boruta analysis in R to identify genes most predictive in estimating expression in the imputed target gene [48]. Confirmed genes were utilized by random forest decision trees to create predictive models for each imputed gene target [40]. Normalized CGGA data was used for training while normalized DSP data was passed through models to predict imputed expression. Any imputed gene was explicitly labeled in the analyses.

### Statistical testing

Statistical evaluation of clustering distribution was measured using chi-square testing. Boxplot comparisons were statistically validated through initial screening by one-way ANOVA and specific group comparisons using Welch’s t-test in R. Due to mixed effects in admixed studies however, intersected disease and histology grouping were assessed using linear mixed effect modeling through the lme4 package in R to measure effect significance through mixed ANOVA [49]. Post-hoc significance testing for mixed group comparisons were performed using the emmeans package in R with Bonferroni *p*-value correction [50].

### List of R packages

Primarily used R packages were Rtsne; umap; fpc; dplyr; factoextra; e1071; caret; cluster; tidyverse; reshape2; Boruta; randomForest; survival; survminer; ggplot2; ggpubr; dbscan; WGCNA; EnhancedVolcano; reticulate; lme4; lmerTest; sjstats; emmeans; org.Hs.eg.db; pheatmap; enrichplot; clusterProfiler; EBImage; CRImage. Full listing of R packages and workflows are available at: https://github.com/WesleyWang913/Wang_et_al_2023_Acta_Neuro_Comms_Code

## Results

### Cancer-immune activity play critical roles in the differential outcome of patients experiencing recurrent glioblastoma

**Fig. 1:**
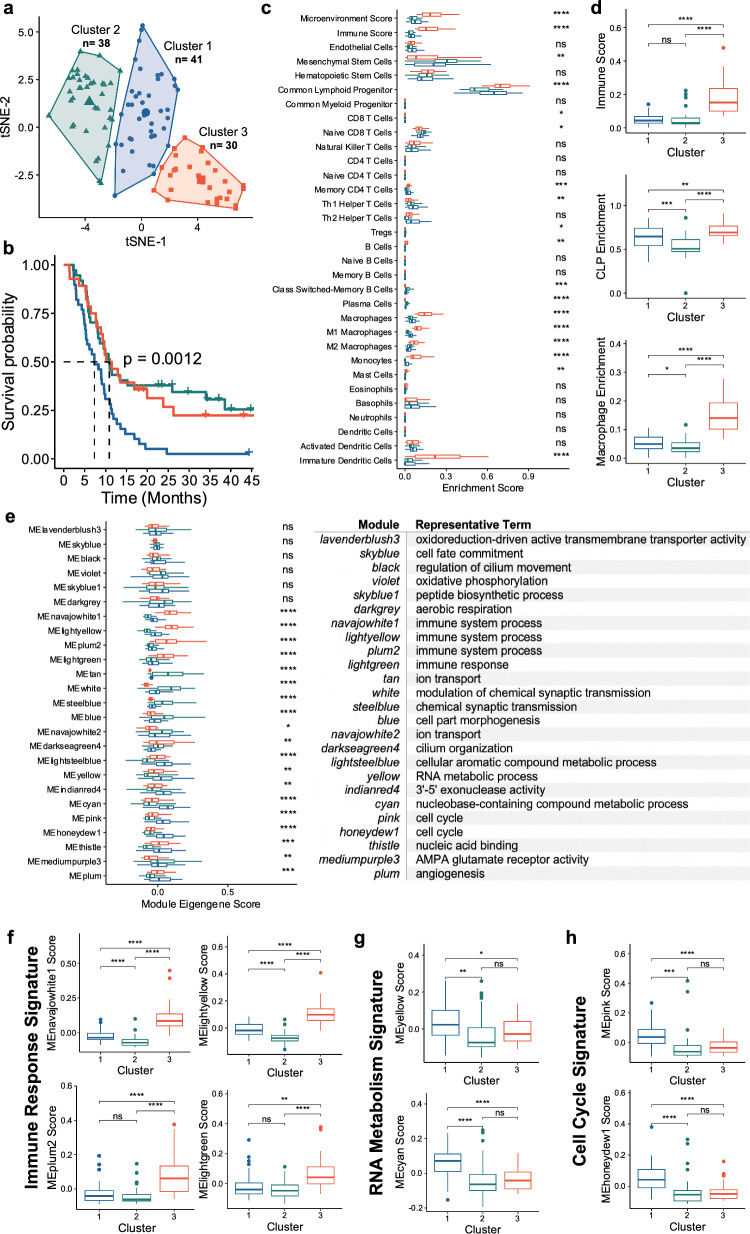
Progressive GB clinical outcomes are driven by variations in tumor immune response and cancer cell replication. (**a**) kMeans clustering of recurrent GB samples based on select gene expression profile. (**b**) KM curve survival of patients based on cluster. (**c**) xCell based cell enrichment scores of clusters. (**d**) Term specific xCell enrichments. (**e**) WGCNA module eigengene score and representative term based on cluster. (**f**–**h**) Process-specific eigengene scores. Initial comparisons were screened by ANOVA and *t* tests were used for specific groupings: *p ≤ 0.05, **p ≤ 0.01, ***p ≤ 0.001, ****p ≤ 0.0001

*Status quo* pathologic workflows assess second resections for presence of atypical cells concerning for proliferative cancer growth and measuring abundances of immune cells and necrosis. We sought to first validate the influence of proliferative stem cell and immune cell activity on survival outcomes in patients experiencing glioblastoma recurrence following primary surgical management with chemoRT. To achieve this, we clustered pathologically confirmed recurrent GB RNAseq samples from the CGGA using an 83-gene signature matrix based on current literature to identify whether (i) recurrent glioblastoma patients clustered into gene signature-defined subpopulations and (ii) determine the extent to which such subpopulations would exhibit differential survival activity (Additional file 1: Table S1; S11). kMeans clustering grouped patients into 3 subpopulations (Fig. 1a). We validated the statistical significance of these groupings by measuring the within cluster distance and between cluster distance of our actual dataset against a scrambled randomized dataset (Additional file 1: Fig. S4; *p* < 0.001). Kaplan–Meier analysis demonstrated overall patient survival among clusters significantly varied (Fig. 1b). Patients in CGGA cluster 1 (blue) exhibited significantly poorer survival outcomes to those in CGGA clusters 2 and 3 (green and red respectively) (*p* = 0.0012). Specifically, an approximately 5-month median survival difference was present between CGGA cluster 1 and CGGA clusters 2 & 3 [dotted line]. These results highlight that specific gene signatures related to immune cell activity and cancer cell proliferation both (i) cluster recurrent glioblastoma patients into distinct subpopulations which (ii) exhibit differential survival activity.

While our previous results highlighted the validity of applying proliferative stem cell and immune cell markers to stratify patients, we wished to better understand the driving processes behind CGGA cluster 1’s poor survival and evaluate whether other concurrent biologic processes or molecular functions not represented in our 83-gene signature were varied. All assessed genes [n = 23,987] were passed through both xCell and WGCNA to assess for processes not highlighted in the original 83-gene signature analysis. With respect to immune enrichment, T-cells, myeloid cells, and lymphoid progenitors varied amongst clusters (Fig. 1c). Specifically, significant enrichment in the overall immune score was present in CGGA cluster 3 when compared to either CGGA clusters 1 or 2. Moreover, common lymphoid progenitors (CLPs) or macrophages were most elevated in CGGA cluster 3, but cluster 1 did show a slight increase in enrichment to cluster 2 (Fig. 1d). Taken together, these data demonstrate that CGGA cluster 3 shows strong immune involvement in the tumor microenvironment, with CGGA cluster 1 showing some immune enrichment to a lesser extent but still greater than cluster 2—highlighting a reduced, but active immune involvement in our poor surviving patients. We conclude that these three clusters of glioblastoma patients show differential survival and differential immune cell deconvolution.

With respect to other biologic processes however, WGCNA was used to group genes into color modules based on similar co-expression patterns and related to biologic processes or molecular functions using gene ontology. Quantitatively, module expression is represented by an eigengene value taken from the first principal component of all genes within a module. Through this approach, relative elevation of an eigengene score based on a clinical phenotype against compared groupings signifies enrichment for the represented process among the differentially regulated genes. Amongst those with significant GO enrichment, all modules listed below *navajowhite1* showed significant variation across clusters (Fig. 1e). Notably, modules related to immune response [*navajowhite1, lightyellow, plum2, lightgreen*] consistently showed the highest eigenscore in CGGA cluster 3 (Fig. 1f). However, RNA/nucleobase metabolism [*yellow, cyan*] or cell cycle activity [*pink, honeydew1*] showed highest elevation in eigenscore in cluster 1 (Fig. 1g, h). It was additionally noted that when assessing overlapped genes related to lymphocyte (GO:0046651) or macrophage (GO:0061517) proliferation, genes represented in our cell cycle color modules showed little overlap while immune process color modules contained several immune proliferative markers (Additional file 1: Fig. S5). Based on these observations, it became more apparent that while cluster 2 & 3 spatially varied in the tSNE plot, the stratification was associated with immune-based signatures but did not correlate with differential survival between these 2 CGGA clusters. However, in the case of cluster 1 which showed the poorest mean overall survival, while some degree of immune activity was evidenced in the analyses, the strongest processes associated in this group included cell cycle activity—as captured in our 83-gene signature—but also active cellular metabolism. We conclude that cluster 1 recurrent glioblastoma patients show enrichment in cellular proliferation and biosynthesis pathways.

### Glioblastoma progression and pseudoprogression can be largely stratified by overall cellular proliferation or immune response present in novel lesions

**Fig. 2:**
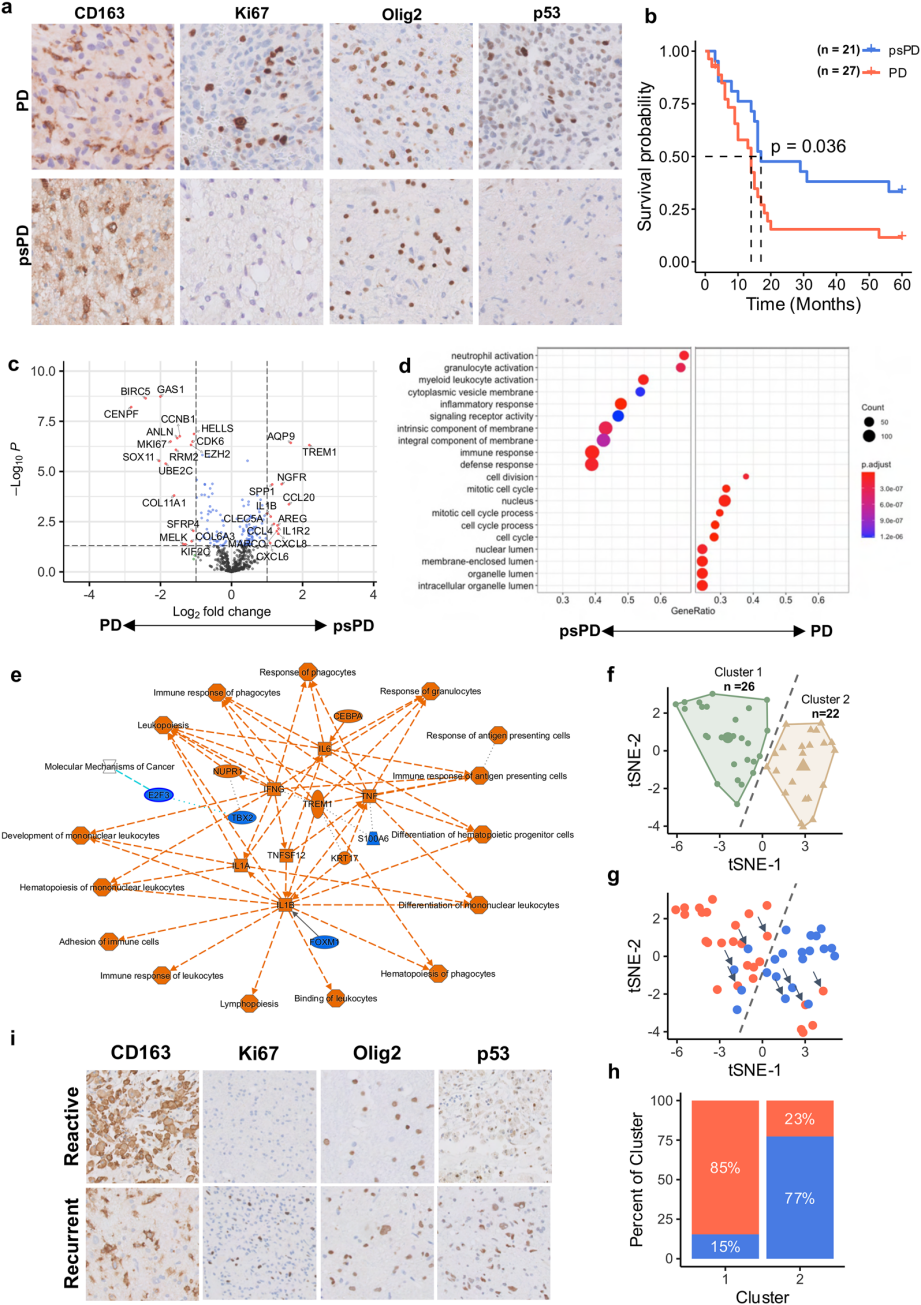
Immune and proliferative stem cell-related signatures accurately distinguish PD and psPD events. (**a**) IHC representation of pathology utilized to best differentiate PD and psPD. (**b**) KM survival of GB patients based on PD (n = 27) vs psPD (n = 21) designation following second surgery. (**c**) Volcano plot differential gene expression of cancer signature based on PD (left) and psPD (right). (**d**) Gene ontology representation of enriched psPD (left) and PD (right) pathways from differential analysis. (**e**) IPA network summary of enriched pathways for PD (blue) and psPD (orange) with driving markers based on differential expression analysis. (**f**) Unsupervised kMeans clustering of samples based on top 150 DEGs from bulk analysis. (**g**) Enhancement status pseudo-coloring of PD (red) and psPD (blue) in previously clustered cases. Arrows represent samples showing admixed pathology taken for spatial analysis. (**h**) Distribution of clinical diagnosis within clusters identified in tSNE map. (**i**) Representative IHC imaging from single case highlighting areas of atypical cellularity (recurrent) and immune infiltration (reactive) within a single lesion. Chi-square: X.^2^ = 16.11, *p* = 5.95e-05

The neuropathological workflows deployed for the diagnosis of PD versus psPD has been highly debated within the community. The main objective of the neuropathological evaluation is to determine the extent to which novel contrast enhancing lesions are due to reactive changes or neoplastic processes. The workflow performed at our institution is highlighted in Fig. 2a. Although our CGGA findings support that differential survival outcomes are present by assessing cell cycle, metabolism, and immune response signatures in novel lesions, psPD involvement was not assessed within the dataset. Turning to samples retrospectively assessed at James Cancer Center/OSU, we sought to both (i) confirm the efficacy of our disease management of novel enhancement lesions by comparing survival outcomes of patients defined as psPD or PD by the neuro-oncologists, and (ii) objectively assess whether cases reviewed by neuropathology could be stratified by cell cycle, metabolism, and immune response signatures molecularly.

As neuropathological guidelines for assessing PD and psPD are ill-defined, final diagnosis by neuro-oncology was used to set our ground truth to group our pathologic specimens. Comparing patient samples used in our accompanying molecular studies, patients designated as psPD showed significantly higher median survival times nearing 5 months following second surgery—with more patients designated as psPD surviving during the duration of the study (Fig. 2b; *p* = 0.036). Although retrospective, these results highlight the point that using our current neuropathology diagnostic approach in stratifying patients does capture patients who are likely experiencing psPD and who have more favorable prognoses. To compare processes separating PD from psPD (as defined by clinical neuro-oncology), 48 samples were evaluated by bulk gene expression using the nCounter PanCancer360 assay—a gene expression test composed of probes that quantify cancer proliferative and/or immune signature-related genes. This assay’s compatibility with FFPE tissue permits incorporation into current neuropathologic workflows. Differential expression analysis highlighted proliferative/stem cell markers including *MKI67* and *Sox11* in the direction of PD, while immune signaling markers such as *CCL20, ILR2,* and *CXCL8* were in the direction of psPD (Fig. 2c). In psPD patients, GO terms highlighted several processes focused on neutrophil/myeloid activation and immune signaling. In contrast, GO terms found in PD patients focused on cell division and cell cycle activity (Fig. 2d). This is further recapitulated in ingenuity pathway analysis (IPA), as enriched terms to psPD represent several immune response pathways driven by *TREM1, TNF, IFNG, IL1,* and *IL6* while PD was primarily demarcated by *E2F*-mediated cancer activity (Fig. 2e). Together, these results highlight the capacity of stratifying PD and psPD using traditional immunohistochemistry (IHC) approaches by focusing on pan-immune activation in psPD events.

To better assess the predictive capability of these markers, top 150 differentially expressed genes (DEGs) were extracted and used to cluster samples in an unsupervised fashion based on their gene expression profile as measured by the nCounter assay (Additional file 1: Fig. S2). Overall, kMeans analysis identified 2 statistically significant clusters (Fig. 2f; Additional file 1: Fig. S6; *p* < 0.001). Distribution of PD and psPD related to cluster identity with PD cases representing 85% of cluster 1 and psPD cases representing 77% of cluster 2 (Fig. 2g, h; X^2^ = 16.11, *p* = 5.95e-05). This *in-silico* analysis furthers the point that such stem cell proliferative/immune signatures hold promise in group stratification. However, for cases which clustered near/in the opposing side, it was noted that samples displayed a mixed tissue histology of both immune infiltration and concerning cancer stem cell-like proliferation (Fig. 2i). Thus, intra-lesion heterogeneity may underscore the failure of our clustering to fully stratify both populations. In conclusion, stratification of novel enhancing lesions using cancer-immune signatures successfully stratifies most PD and psPD cases; however, a rarer subset of patients does not fit this approach due to high levels of tissue heterogeneity that obfuscates bulk gene expression analyses.

### Immune infiltration abundance and cell morphology separate PD and psPD

**Fig. 3:**
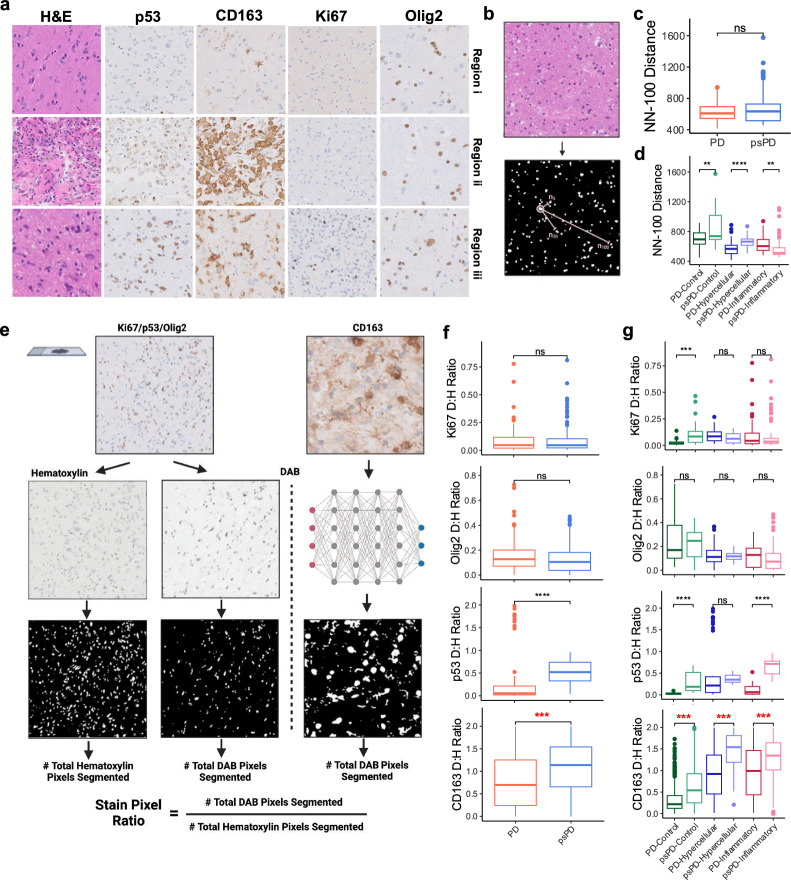
Abundant immune infiltration is preserved in psPD events in spite of admixed presentation. (**a**) Representative 40 × histology of captured regions of interest showing admixed presentation in a sample. (**b**) Nearest-neighbor distance schematic of segmented nuclei from hematoxylin layer of H&E images among (**c**) novel enhancement status and (**d**) status sub-stratified by tissue histology. (**e**) DAB to hematoxylin staining ratio calculation schematic using image thresholding for Ki67, p53, and Olig2 while CNN-based segmentation was applied for CD163. Staining ratios among (**f**) novel enhancement status and (**g**) status sub-stratified by tissue histology. *t* test: **p ≤ 0.01, ***p ≤ 0.001, ****p ≤ 0.0001. Mixed effect modeling was additionally performed with post-hoc *p* value correction. All comparisons in bolded red represent preserved significance with mixed effect correction

In our experience, up to a third of cases present as a mix of treatment effect and tumor which may lead to discordant clinical-pathological correlation [6]. We therefore sought to identify how well our histological approach to stratifying PD and psPD could be identified in specimens that showed marked morphological heterogeneity. To do so, eight samples were selected with tSNE map location in areas where both PD and psPD cases sat in close proximity—highlighting similar expressional profiles (Fig. 2g; arrows). All samples were additionally pathologically confirmed to show mixed presentation and collected for representative H&E, p53, CD163, Ki67, and Olig2 imaging in regions defined by neuropathologist observers as (i) “control” brain (characterized by quasi-normocellular gray or white matter), (ii) “inflammatory” immune reactive brain (characterized by hypercellular gray or white matter without significant neoplastic cells noted on the morphological and immunohistochemical biomarker workup), and (iii) “hypercellular” recurrent cellularity brain within a sample (defined as hypercellular gray or white matter showing neoplastic cells based on the morphological and immunohistochemical biomarker workup) (Fig. 3a; Additional file 1: Table S2). While all samples showed mixed presentation, a ground truth PD or psPD status was designated based on retrospective neuro-oncologic diagnosis [quoted name of regions (“control”, “inflammatory”, and “hypercellular”) will be used to distinguish histology against novel enhancement status outcome (PD, psPD) from here on].

We quantified the histologic variation in 8 samples showing heterogenous histological architectures within our workflow by measuring (i) overall tissue cellularity, (ii) IHC staining used in the original neuropathological workflow, and (iii) cell morphology variation based upon ground truth clinico-pathologic designation using automated image analysis workflows. To compare the overall cellularity present between PD and psPD events, H&E images were assessed using a nearest neighbor distance calculation. This approach was accomplished through automated image deconvolution and segmentation of hematoxylin^+^ nuclei to calculate mean distance of a nuclei from its hundredth nearest neighbor (Fig. 3b). It was found that the mean 100th nearest neighbor distance of segmentations did not significantly vary between PD and psPD events (Fig. 3c). However, when stratified by histology, PD events showed significantly reduced distances in “control” and “hypercellular” regions, while psPD showed reduced distance in “inflammatory” regions (Fig. 3d). It was important to however note the expansion of sample size due to replicates for histologic regions. In consequence, linear mixed effect modeling was performed to control for replicate bias and measure the effect of both our disease status and combined disease status plus histology subtype groupings to our measured outcome. Mixed effect ANOVA highlighted no significant effect of enhancement status to nearest neighbor distance [*p* = 0.65, η^2^ = 0.04], but a significant effect when enhancement status and histology were evaluated together [*p* = 0.03, η^2^ = 0.03] (Additional file 1: Table S3). Due to the small effect however, post-hoc testing for mixed status and histology groupings did not see preservation of significance in Fig. 3d. Our findings thus suggested that overall presentation of cellularity between PD and psPD events did not vary between groups due to differential cellularity in histologic regions in mixed samples.

We next quantified staining in our samples using a DAB to hematoxylin pixel ratio calculation (Fig. 3e). Total DAB^+^ pixels were divided by hematoxylin^+^ pixels in an image to generate a staining ratio which would control for variable cellularity between collected images and pixel size of images. Notably, neither Ki67 nor Olig2 showed significant differences in staining ratio between novel enhancement statuses; however, both p53 and CD163 showed increased staining ratio towards psPD (Fig. 3f). Staining ratio was additionally assessed between histologic subtypes (Fig. 3g). Neither Ki67 nor Olig2 were found to show significant differences in IHC staining in “inflammatory” and “hypercellular” regions. Moreover, mixed effect ANOVA showed no significant effect on staining ratio for status or combined status plus histology groupings in Ki67 and Olig2 (Additional file 1: Table S3). In contrast, p53 showed significant elevations in “control” and “inflammatory” regions, but not in “hypercellular” sites where the stain is applied to evaluate cancer cell mutations. Moreso, enhancement status showed no significant effect on p53 staining ratio [*p* = 0.2, η^2^ = 0.26], but significant effect by status and histology [*p* = 1.4e-5, η^2^ = 0.09] (Additional file 1: Table S3). Assessment of histology however showed p53 staining in absence of nuclei with atypical Olig2 staining and Ki67 staining (Additional file 1: Fig. S7). In contrast, both PD/psPD status and combined status with histology showed significant effect on CD163 staining ratio [Status-alone: *p* = 2.2e-16, η^2^ = 0.44; Status-Histology: *p* = 2.2e-16, η^2^ = 0.02]. A strong effect of enhancement status to CD163 staining was thus highlighted as CD163 was shown to have significant staining elevation among all histologic subtypes in the direction of psPD. Post-hoc testing additionally found significance was only preserved between pairwise groupings in CD163 [Bonferroni corrected *p* < 0.0001 in all groupings] (Fig. 3f, g). Thus, while the dominant staining of CD163 was present in psPD events, we conclude that standard IHC biomarkers are minimally effective to confidently claim a lesion as psPD.

**Fig. 4:**
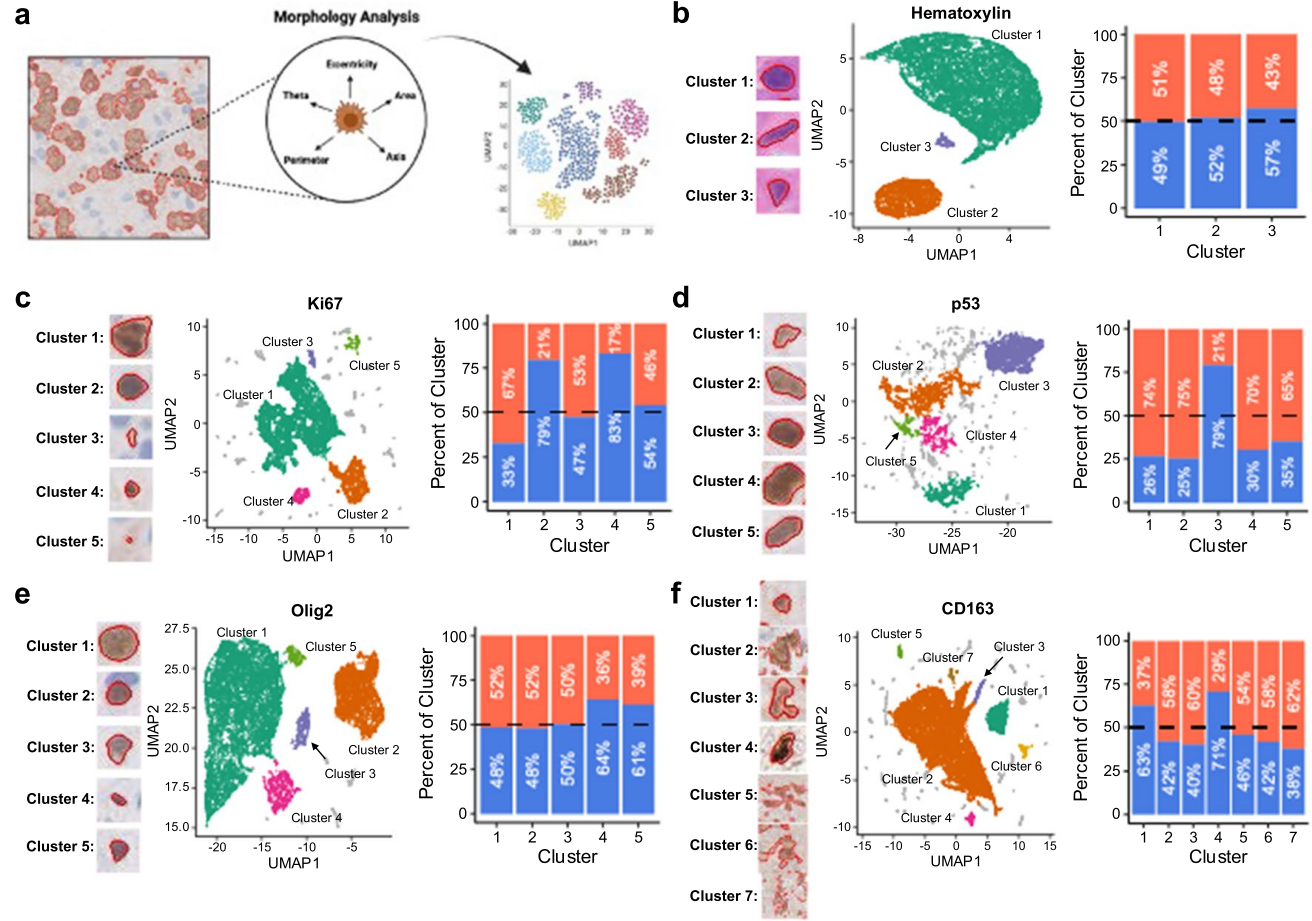
UMAP clustering of IHC stained cell morphology. (**a**) UMAPs of segmented cells based upon cell morphology features with accompanying cluster distribution of segmentations from PD (red) and psPD (blue) events for (**b**) hematoxylin, (**c**) Ki67, (**d**) p53, (**e**) Olig2, and (**f**) CD163. Representative cell morphology for each cluster is shown to the right of UMAPs. Expected even distribution of diagnosis is represented by the dotted line. Chi-square.: Hematoxylin: X^2^ = 11.88, *p* = 0.0026; Ki67: X^2^ = 1828.8, *p* < 2.2e-16; p53: X^2^ = 2136.8, *p* < 2.2e-16; Olig2: X^2^ = 107.59, *p* < 2.2e-16; CD163: X.^2^ = 399.13, *p* < 2.2e-16

It was additionally apparent that morphologic variability was present across stains (Additional file 1: Fig. S7). We assessed morphologic variation within stains by generating cellular clusters based on cell morphology-extracted features and measured whether the distribution of these morphologic subpopulations varied between PD and psPD. We accomplished this by evenly extracting 16,000 segmented cells from both PD and psPD events from hematoxylin and other IHC stains to cluster samples based on their cell morphology features using UMAP (Fig. 4a). Across stains, multiple clusters were generated with variation from an even distribution (Fig. 4; dotted line). Interestingly, across all stains we found significant distribution changes based on chi-square analysis [Hematoxylin: X^2^ = 11.88, *p* = 0.0026; Ki67: X^2^ = 1828.8, *p* < 2.2e-16; p53: X^2^ = 2136.8, *p* < 2.2e-16; Olig2: X^2^ = 107.59, *p* < 2.2e-16; CD163: X^2^ = 399.13, *p* < 2.2e-16], but the degree of deviation from even distribution varied. Within hematoxylin^+^ segmentations, only 3 clusters were identified with the majority of nuclei falling into cluster 1 (large round nuclei) and 2 (slim ellipsoid nuclei). Additionally, a slight increase of psPD distribution was represented in cluster 3 which represented smaller nuclei with variable shape (Fig. 4b). Similarly, within Olig2^+^ segmentations, while several clusters were identified the predominance of nuclei fell within clusters 1 and 2 (round nuclei reminiscent of oligodendrocytes with variable size) which showed close-to even distribution. However, two subpopulations in cluster 4 and 5 representing small, variably shaped nuclei were shown to have slight increase in psPD distribution as well (Fig. 4e). In contrast, Ki67, p53, and CD163 generated several clusters with many smaller clusters omitted due to the rarity of segmentations present in those groups. Within Ki67^+^ segmentations, the majority of nuclei grouped into cluster 1 (large, abnormally shaped nuclei reminiscent of cancer cells) which predominately was composed by PD samples. However, both cluster 2 and 4 (smaller, round nuclei morphology reminiscent of immune cells) showed a heavy predominance from psPD events (Fig. 4c). In p53^+^ segmentations however, distribution of several clusters (clusters 1, 2, 4, and 5) were all shown to be primarily from PD related events with highly irregular morphology reminiscent of cancer cells. Only cluster 3 (rounder nuclei morphology) was observed to predominately originate from psPD events (Fig. 4d). Finally in CD163^+^ segmentations, the predominance of cells were found in cluster 2 (large branching cells with highly variable shape) with a slight predominance toward PD; moreover, smaller clusters from 3, 5, 6, and 7 showed similar distributions with overall similar morphology to cluster 2. However, other small clusters (1 and 4) showed stronger predominance of psPD with morphology showing smaller punctate staining with variable shape (Fig. 4f). Overall, hematoxylin was observed to have the least morphologic clustering while other IHC stains generated several morphologic subclusters. Interestingly though, within our analysis it was seen that several clusters—particularly those from Ki67 and p53—showed subpopulations of nuclei with distinct morphologic features that more predominated in PD or psPD events. In consequence, while the overall staining of IHC markers targeting proliferative cancer cells was not significantly varied between novel enhancing events, morphology of segmented cells may help identify subpopulations that vary between groups. While these findings support the ability to stratify populations using our current histology workflow, the challenge for a pathologist will likely increase due to the high background staining found within CD163 coupled with the need to annotate rare morphology to accurately find landmarks representative of PD and psPD.

### Cancer immune activity varies in PD and psPD events despite admixed histology

**Fig. 5:**
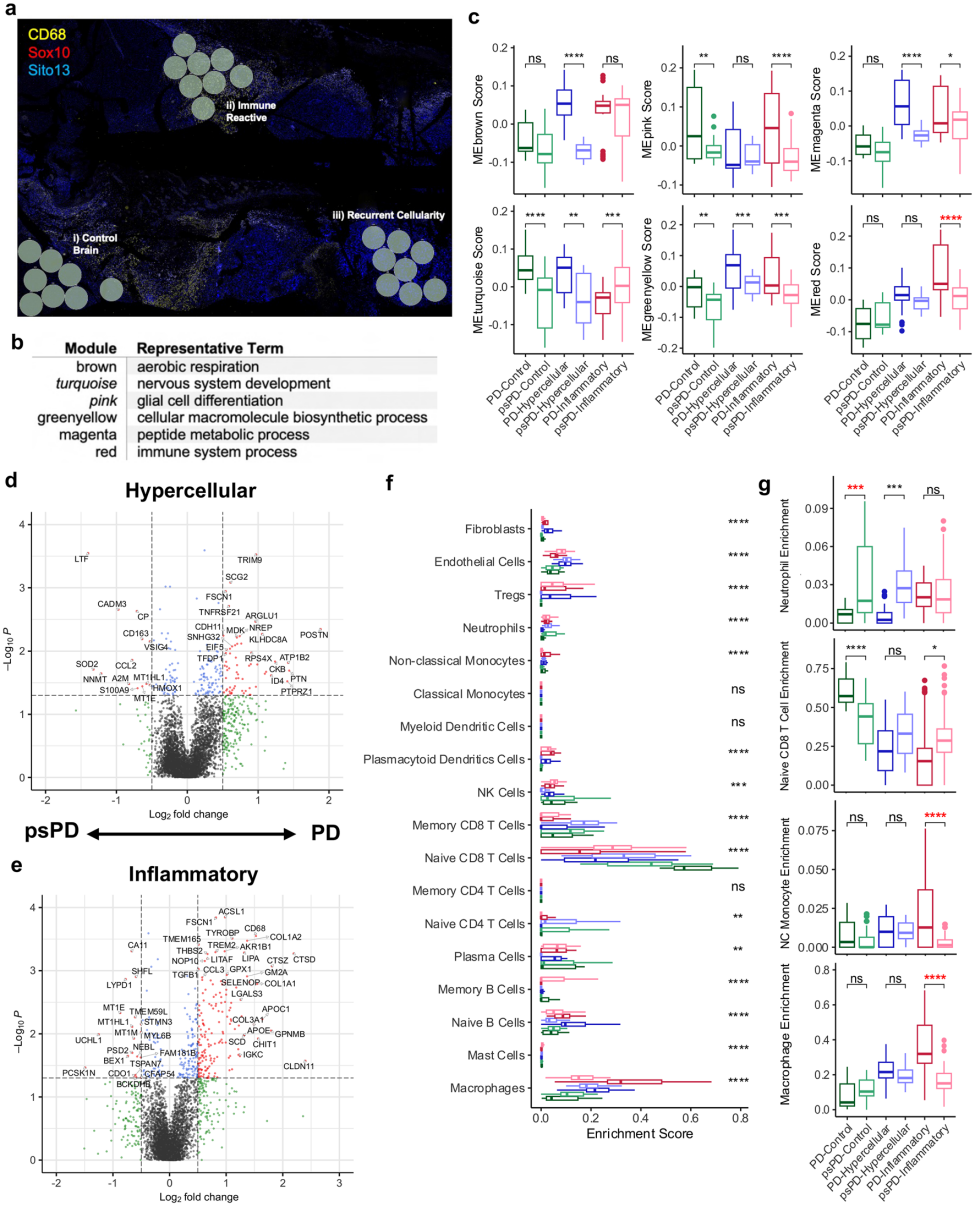
Admixed novel enhancement lesions are stratified by immune cell subtype infiltration. (**a**) Representative capture schematic of GeoMx machine. Gray circles represent locations of RoI collection. (**b**) Representative enriched GO term in WGCNA color modules with (**c**) eigengene score compared across histologic subtypes. Differential analysis of PD and psPD events with respect to (**d**) “hypercellular” and (**e**) “inflammatory” histology. (**f**) Overall immune population comparison among stratified groups. (**g**) Specific boxplot comparisons of clusters for neutrophil enrichment, naïve CD8 T-cell enrichment, non-classical monocyte enrichment, and macrophage enrichment. Initial comparisons were screened by ANOVA and *t* tests were used for specific groupings: *p ≤ 0.05, **p ≤ 0.01, ***p ≤ 0.001, ****p ≤ 0.0001. Mixed effect modeling was additionally performed with post-hoc *p* value correction. All comparisons in bolded red represent preserved significance with mixed effect correction

Although our image analysis found the preserved efficacy of CD163 immune abundance and use of morphologic variation to stratify novel enhancing lesions, the inability to stratify whether cancer cells present in tissue are proliferating prevent a pathologist from confidently claiming a lesion as psPD. We sought to identify more objective differences between PD and psPD related samples by assessing the spatial variability of expression signatures with respect to the histologic regions shown in Fig. 3a to uncover novel molecular changes across histology. Using the same 8 mixed samples from our image analysis, we immuno-fluorescently labeled slides to identify areas of immune infiltration (CD68^+^) or stem cell presence (Sox10^+^) coupled with overlayed clinical IHC imaging to generate RoIs in “control”, “inflammatory”, and “hypercellular” histology regions (Fig. 5a; gray circles).

WGCNA assessed whether known or novel biologic pathways or molecular functions varied between groups and were consistently changed irrespective of histology. It was seen the primary processes which varied amongst sampled regions included nerve/glial cell development (*turquoise, pink*), biosynthesis/metabolism (*greenyellow, magenta, brown*), and immune processes (*red*) (Fig. 5b). Specifically, nervous system development related modules significantly increased in PD events compared to psPD event in “control” and “hypercellular” regions within the *turquoise* module. Additionally, “inflammatory” regions displayed increased eigenscore in the *pink* module for PD (Fig. 5c). Overall, these findings support true PD processes show increased cellular differentiation with particular regard to glial populations (*pink*) in not only “hypercellular” regions concerning of cancer recurrence, but also sites which may appear as more normal “control” brain or largely “inflammatory” under histology. This point is furthered when evaluating more biosynthetic/metabolically relevant modules, with particular regard to the *greenyellow* module. Irrespective of histology, PD had significant increased eigenscore for cellular biosynthesis (*greenyellow*) (Fig. 5c). Interestingly, converse elevation in immune activities (*red*) was not significantly varied in either “control” or “hypercellular” histology and, in fact, significantly elevated in the PD cases for “inflammatory” histology (Fig. 5c). Mixed effect ANOVA demonstrated across color modules that no significant main effect on eigengene score was seen with status alone, but significant effect was present with status and histology combined (Additional file 1: Table S4). Post-hoc testing however found that several pair-wise groupings did not retain significance, but magenta module eigengene differences in “hypercellular” regions between PD and psPD approached significance [Bonferroni corrected *p* = 0.07] and red module eigengene differences in “inflammatory” regions remained significant [Bonferroni corrected *p* = 0.005] (Fig. 5c). Taking these observations, elevations in biosynthetic activity in “hypercellular” regions for PD trended but were limited by sample size. Elevation in immune system processes for PD in the inflammatory region may indicate fundamentally different immune pathways present in psPD inflammatory regions.

As WGCNA did not show a global elevation in immune response within psPD events across histology, we posited that the lack of congruency between our findings may be due to differential infiltration of immune cells between groups. Although *CD163* was noted to be significantly enriched in “hypercellular” events towards psPD, the log change was relatively low (Fig. 5d). In contrast, markers of immunosuppression polarization in myeloid cells were observed to be enriched in “inflammatory” PD events including *TREM2, APOC1, APOE,* and *LGALS3* (Fig. 5e; [51]). “Control” regions showed fewer significantly enriched genes, but neuronal markers such as *ENO2* were seen elevated towards PD (Additional file 1: Fig. S8). To examine this further, immune deconvolution was performed over the dataset to evaluate predicted immune infiltration across these groupings (Fig. 5f). Particular variation was present among neutrophil, monocyte/macrophage, and CD8 T-cell populations (Fig. 5g). To be specific, both “control” and “hypercellular” regions had elevated neutrophil enrichment in psPD cases when compared to PD cases of matching histology. Furthermore, “inflammatory” regions showed enrichment of CD8 T-cells in psPD events, while enrichment for monocyte/macrophage populations was elevated in PD events. Mixed effect ANOVA additionally found that enhancement status alone did not have significant effect on immune enrichment, but significant effect was present in combined status and histology groupings in our particular cell populations (Additional file 1: Table S4). Post-hoc testing highlighted mixed effect-corrected significance was maintained in neutrophils in “control” regions [Bonferroni corrected *p* = 0.047], non-classical monocytes in “inflammatory” regions [Bonferroni corrected *p* = 0.024], and macrophages in “inflammatory” regions [Bonferroni corrected *p* = 0.0002] for Fig. 5g, Overall, while a global elevation in immune response was not seen across histology for psPD, psPD displayed elevations of certain cellular population such as neutrophils amongst specific histologic regions. Moreover, while a surprising elevation in immune response was detected in PD events, our results highlighted a domination of myeloid enrichment with biomarkers associated with pro-tumoral polarization. In summary, despite the blunted efficacy of our cancer-immune signatures in admixed tissue samples, significant variation in immune microenvironment activity in spite of similar histology is present between PD and psPD.

## Discussion

### Histologic evaluation of cancer stem cell and immune activity is representative of the molecular features defining PD and psPD

Our approach to assessing novel enhancing lesions hinges on the validity of traditional approaches. Specifically, that histopathological classification of PD may be rendered by identification of irregularly shaped Olig2^+^ nuclei and with active proliferation noted by Ki67 immunohistochemistry. The scientific premise for this traditional approach is based on prior reports indicating high Ki67 expression levels in PD compared to psPD [52]. Furthermore, the regeneration and evolution of GB has been well linked to the functional activity of cancer stem cells (CSCs) that remain present as rare populations following surgical resection [53]. In our own data, our initial RNAseq studies showed a poorer survival outcome in patients which were separated based primarily on the elevation of both cell cycle and cell metabolic processes. Moreover, these proliferative differences were seen to not overlap with immune proliferative activity that was better represented in our immune process modules. This association may thus underlie the poor survival outcomes seen in our PD patients as the predominant GO terms enriched in PD all related to cell cycle activity in our bulk RNA analysis. Additionally, upregulation of *MKI67* and *Sox11* in our differential expression bulk RNA analysis further recapitulates the utility of assessing stem cell proliferation using Ki67 and Olig2 IHC to define PD. However, careful interpretation should be noted as Ki67 does not specifically highlight CSC-like populations and our analyses evidenced an additional domination of immune cell activation with psPD which may similarly stain Ki67—warranting the need for IHC morphologic assessment.psPD lesions should expectedly be absent of such concerning cellularity. As the cause of novel enhancement is due to permeability of the blood brain barrier (BBB), increased inflammation following leukocyte recruitment is a predicted component in the pathogenesis of disease [54]. Combined with the broader notion of increased immune abundance with psPD, our approach to psPD assessment was through identification of broad immune infiltration via CD163. The primary finding in our nCounter studies showed a complete domination of terms related to activation of immune populations including macrophages and neutrophils. These results thus substantiate the histologic concept of immune abundance as the key expression signature of psPD. Additionally, our highest differentially expressed probes in the direction of psPD include several chemokines (*CXCL6, CXCL8,* and *CCL20*), but also *AQP9* and *TREM1* which have been evidenced to be represented in glioblastoma by enrichment of AQP9^+^ leukocyte infiltration and TREM-1^+^ myeloid population enrichment in GB peri-necrotic zones [55, 56]. In consequence, this activity may be indicatory of the robust immune infiltrative sequelae caused by recruitment of leukocytes into the lesion leading to downstream BBB breakdown and chemokine cascade.

### Automated morphology characterization can assist in the stratification of admixed novel enhancement lesions histologically

In our *in-silico* study, the effectiveness of stratifying patients using cancer immune signatures was highlighted by the overlap of our unsupervised nCounter clusters to the distribution of PD and psPD events. However, it must be noted that a smaller subset of cases (9/48 cases) did not fit this model. Melguizo-Gavilanes et al. had found that in cases where histology was discordant with radiologic/clinical diagnosis a large proportion of second surgery samples show mixed presentation [6]. In the first part of our image analysis studies, CD163 was found to broadly stain confirmed psPD lesions irrespective of histologic subtypes we identified. These findings thus further support the previous notion that psPD represents a hyperimmune state presenting with broad immune abundance in tissue [57]. However, abundant CD163 staining cannot rule out cancer recurrence if areas of Olig2^+^ nuclear atypia with notable Ki67 positivity are present. As these sites raise primary concern for CSC-like populations, CSC subpopulations have been noted to have extremely robust tumorigenicity and self-renewing capability to regenerate cancer [12]. In consequence, even if such areas represent rare populations in a histologic section, careful consideration must still be given to decide whether therapy plan changes are warranted. The evaluation of histology is not limited by stain positivity alone. Cellularity, location, and morphology all may be evaluated by a pathologist with novel tools from computational pathology when generating a differential diagnosis. Both residential immune populations and CSCs have been found to have a spectrum of morphological states which act as proxies to transcriptional changes, but the clinical application of these findings are difficult due to observer subjectivity [58–60]. In consequence, our morphology UMAP clustering computational pathology workflow both reduces the subjectivity of assessing cell morphology and helps to identify morphologic subpopulations that an individual may integrate into their workflow. Notably, Ki67, p53, and CD163 were shown to have several morphologically derived clusters which showed differential distribution of PD and psPD events. Raising potential morphologic markers pathologists may integrate in their differential assessment. While further validation is needed, this result raises the potential application of machine learning-based image analysis workflows to supplement clinical decision making. Aida et al. predicted *Nanog* expression could be estimated from morphological subtypes of CSCs using CNNs [60]. Myeloid populations additionally have been commented in GB to have dynamic morphologic change which underscores shifts in immune activation [59, 61]. In parallel, the identification of morphologic features from our automated image analysis pipeline can be used against our spatially derived molecular profile to better predict active CSC metabolic states or immune activation states morphologically.

### Molecular enrichments support exploration of IHC stains which evaluate cancer metabolism and immune cell polarization in novel enhancing lesions

Our current neuropathologic framework focuses on the notion that PD and psPD can be broadly dissociated by cancer cell proliferation and immune activation using a select series of IHC markers. However, this approach can fail if overlapping immune activation or proliferation is present. Cell proliferation was notably the largest component in our workflow which failed to stratify between mixed presenting lesions based upon Ki67 staining ratio. Moreso, in our DSP WGCNA, cell cycle/division related enrichments were not observed as seen in our bulk analysis. As Ki67 does not exclusively label proliferating cancer cells, our stain may be complicated by other benign proliferating cell populations. Nevertheless, cell cycle activity was shown to be globally elevated in PD in our nCounter studies. While limited by sample size, our DSP studies did show a trend of biosynthesis enrichment in “hypercellular” sites—which may underscore active cancer metabolism as seen in our CGGA analysis. Cancer cell populations may be represented by this molecular finding as CSCs have been shown to fluctuate between states of dormancy and proliferation based on the supportiveness for cancer growth by the microenvironment [62, 63]. Growing literature has additionally shown the efficacy of PET imaging to assess cancer metabolism through glucose and amino acid tracer uptake and its ability to accurately stratify PD and psPD populations due to increased metabolic uptake in recurrent disease [54, 64]. Kaya et al. specifically observed in the context of differentiating second surgery lesion samples showing reactive gliosis or GB recurrence, the metabolic marker PTBP1 showed promise in stratifying patients into PD and psPD [65]. Accordingly, potential IHC biomarkers to evaluate cellular metabolism during neuropathologic work up may additionally help to stratify lesions in challenging cases like admixed presentation but expanded studies will be needed to validate these claims.

Conversely, both our bulk analysis and CD163 image analysis highlighted a robust predominance of immune response expression in psPD events that surprisingly seemed to dissipate based on our DSP WGCNA studies. To acknowledge however, the pre-selection of RoIs for specific histology does not accurately capture what is likely seen in our bulk studies. As the assessed “hypercellular” and “inflammatory” histologic regions ultimately influence the decision to diagnose a patient as PD or psPD however, it was critical to identify whether region specific changes in the microenvironment were present. In areas primarily enriched by immune cells marked by a profuse CD163 inflammatory cell infiltrate (“inflammatory” histology), PD events showed enrichment for monocyte/macrophage signatures. These enrichment differences may highlight intrinsic variations in immune polarization in the lesion that separate the 2 entities as differential expression analysis of “inflammatory” regions showed several markers of pro-tumoral myeloid polarization [51]. In conjunction, Giordano et al. assessed the presence of circulating CD163^+^ monocyte populations and observed a phenotype shift of increased monocyte infiltration in GB cases with residual tumor while such populations were almost absent in psPD patients [66]. This observation is similarly shown in our study as monocyte enrichment was highly elevated in our CD163 enriched “inflammatory” sites but highly reduced in psPD. Additionally, immune exhaustion signaling has been implicated in the evolution of GB by both tumor-intrinsic and myeloid cell populations [67–69]. Immune exhaustion as represented by simultaneous *PD1, TIM3,* and *LAG3* elevation was however not observed in PD events using imputed expressional calculation (Additional file 1: Fig. S9). We however noted a reduced presence of CD8-T cells trended in PD events across histologic subtypes aside from “control” histology where the estimated infiltration of myeloid cell populations (macrophages and monocytes) was highly reduced. Taken together, the application of CD163 evaluation for immune reactivity must be interpreted carefully if CSC-like cells identified under histology are present. As CD163 is used to broadly interpret immune presence in tissue, the differences in immune infiltration found in our molecular studies may additionally warrant use of other stains to assess myeloid polarization and its influence on T-cell mediated immune control and cancer growth.

### Supplementary Information

**Additional file 1**. **Table S1.** Clustering gene list for recurrent GB CGGA analysis. **Table S2.** Admixed sample lesion description and pathology. **Table S3.** Mixed effect modeling statistics of image analysis groupings to segmentation measures. **Table S4.** Mixed effect modeling statistics of DSP analysis groupings to WGCNA and immune deconvolution measures. **Figure S1.** Tissue processing framework for molecular studies. **Figure S2.** Top 150 DEGs from OSU PD vs psPD Cases. **Figure S3.** Image processing schematic of IHC stains. **Figure S4.** Cluster statistics of CGGA clustering analysis. **Figure S5.** WGCNA module and immune proliferation gene overlap. **Figure S6.** Cluster statistics of nCounter clustering analysis. **Figure S7.** Representative staining of IHC markers in sub-stratified samples. **Figure S8.** Volcano plot analysis of differentially expressed genes in “control” regions in PD and psPD event from GeoMx. **Figure S9.** Immune exhaustion marker expression in CGGA and DSP datasets.

## Data Availability

Bulk nCounter expressional and GeoMx spatial data are available in GEO under series records GSE231994 and GSE232050 respectively. Image tiles used for image analysis are available from the authors with reasonable request. Full code and packages used for R analysis can be found on https://github.com/WesleyWang913/Wang_et_al_2023_Acta_Neuro_Comms_Code.
