# Supplementary material for: Glioblastoma pseudoprogression and true progression reveal spatially variable transcriptional differences
Source: Acta Neuropathol Commun. 2023 Dec 4;11:192. doi: 10.1186/s40478-023-01587-w (PMC10694987; doi:10.1186/s40478-023-01587-w)

Additional File 1

Table S1: Clustering gene list for recurrent GB CGGA analysis. The table displays the full list of 83 genes used for sample clustering in main figure 1 a. Selected genes were based on previously described immune, stem cell, or proliferative markers associated with cancer growth and tumor immune microenvironment change. The reference paper column shows the relevant publications used as a basis for selecting a marker. Genes are grouped in the process column to the most relevant process highlighted by those markers.

Table S2: Admixed sample lesion description and pathology. Relevant clinical information about the 8 samples used in both our image analysis and DSP studies are shown in the table. All samples were confirmed by pathology to have a mixed presentation as listed in the pathology diagnostic report. Relevant patient protected health information has been removed. Time to enhancement was listed based on nearest months from ChemoRT completion to novel enhancement detection on radiologic imaging. Slide tissue area refers to the area of tissue present on the slide used for DSP analysis. Lesion size was listed by the 2 largest axes measured on clinical report. Ki67% was measured by pathologic interpretation taken from the pathology report. Side, lobe, and diagnosis were all collected from the neuro-oncology report.

Table S3: Mixed effect modeling statistics of image analysis groupings to segmentation measures. Mixed effect ANOVA scores were calculated and reported in the table for novel enhancement status and combined status plus histology. Relevant values for ANOVA score are listed in numerator degrees of freedom (NumDF) versus denominator degrees of freedom (DenDF), F-value, and P-value. Effect size for each grouping is reported by Eta-squared whereby larger scores represent greater effect on the measured outcome. Significance with mixed ANOVA was reported in the main figures as a red asterisk. Calculation of scores was performed in R using linear mixed method calculation to control on random effect due to multiple sample testing.

Table S4: Mixed effect modeling statistics of DSP analysis groupings to WGCNA and immune deconvolution measures. Mixed effect ANOVA scores were calculated and reported in the table for novel enhancement status and combined status plus histology. Relevant values for ANOVA score are listed in numerator degrees of freedom (NumDF) versus denominator degrees of freedom (DenDF), F-value, and P-value. Effect size for each grouping is reported by Eta-squared whereby larger scores represent greater effect on the measured outcome. Significance with mixed ANOVA was reported in the main figures as a red asterisk. Calculation of scores was performed in R using linear mixed method calculation to control on random effect due to multiple sample testing.

Figure S1: Tissue processing framework for molecular studies. FFPE tissues were processed and evaluated using bulk nCounter analysis in the upper path. Tissue was extracted for RNA, measured for RNA integrity and concentration, and processed on a nCounter Pancancer 360 panel. Tissue was sectioned and imaged for GeoMx DSP in the lower path. Tissue was sectioned at 5 microns and stained with conjugated antibodies and probes. Regions of interest were drawn on GeoMx to cleave oligo-tags. Final ground truth designation for binning of cases is based on clinico-pathologic diagnosis rendered by neuro-oncology. Full details of processing can be found in the methods,

Figure S2: Top 150 DEGs from OSU PD vs psPD Cases. Expression heatmap of top 150 DEGs between PD and psPD GB novel enhancement events shown in nCounter studies. Left-side hierarchical clustering had grouped similar expressing genes with names listed on the right. Top-side hierarchical clustering had grouped similar samples with PD in teal or psPD in red. Specific sample label is found on the bottom of the heatmap. kMeans clustering in main figure 2 is independent of the clustering in the heatmap; however, same genes were used in the analysis. Highlighting in either case that not all samples perfectly stratify,

Figure S3: Image processing schematic of IHC stains. (A) Segmentation and analysis scheme for H&E images [n=260 for 8 cases]. Images were deconvoluted and segmented to calculate morphology features. Prior to performing either nearest neighbor spatial distribution calculation or morphology clustering, segmentations were filtered based on size to remove punctate objects. (B) Segmentation and analysis scheme for Olig2, Ki67, and p53 images [n=260 per stain for 8 cases]. Images were deconvoluted and segmented to calculate morphology features. However, a random forest machine classifier was trained to identify segments as true signal or not based on positive and negative controls. This was due to the variable staining intensity across images. After filtration, pixel stain ratio calculation and morphology clustering were done. (C) CD163 scheme for (1) segmentation and analysis of image tiles [n=5,809 for 8 cases] and (2) CNN model training [n=6,524 images]. Development of CNN models was done with an external dataset of CD163 images that were manually annotated by a trained observer. Representation of brown background stain was done by computationally noising the image with brown pixels. After training, images taken from the 8 samples were passed through both models to generate prediction maps of cell processes and bodies. This was done due to the different optimizations needed to accurately identify processes and bodies. Images were then remerged to calculate morphology features and filtered for downstream processing as described in B. (D) Intersection over union accuracy score of CNN models after mask merge against ground truth test dataset [n=1,288]. IoU score was based on accurate pixel categorization of both negative and positive pixels as opposed to an accuracy score. This is based on the fact that accuracy would measure the total number of pixels correctly guessed as positive without integrating negative pixels—causing a bias as most images do not have many positive pixels. Outliers with low score are shown at the bottom of the boxplot—showing discordance of ground truth image and CNN prediction. (E) Representation of outlier segmentations in IoU score showing inaccuracy due to lack of segmentations in ground truth. Ground truths in external dataset were created by a trained observer manually. In consequence, missed segmentations are possible in the ground truth images that the CNN overcame.

Figure S4: Cluster statistics of CGGA clustering analysis. Clustering statistics of (A) average within cluster distance and (B) average between cluster distance of points in actual dataset (green) and randomly scrambled dataset (grey). Randomly scrambled dataset was generated by scrambling values taken from the actual data—effectively eliminating potential relationships in the dataset. Average within cluster distance represents the mean distance a point is from all other points in a cluster, while average between cluster distance represent mean distance of a point from all points in another cluster. Statistically valid clustering would be shown as significant deviation of clustered point scores against a scrambled dataset where no real relationship is present. T-test: **** $p \leq 0.0001$**

Figure S5: WGCNA module and immune proliferation gene overlap. Overlap of genes related to lymphocyte proliferation (GO:0046651) [lower left] or macrophage proliferation (GO:0061517) [lower right] for CGGA WGCNA color modules related to (A) cell cycle activity and (B) immune processes. Gene names were extracted from all found groups and compared using a Venn diagram to identify whether overlapped use of terms was present. In specific, query was done to see whether our color modules contained genes affiliated to immune proliferation,

Figure S6: Cluster statistics of nCounter clustering analysis. Clustering statistics of (A) average within cluster distance and (B) average between cluster distance of points in actual dataset (green) and randomly scrambled dataset (grey). Randomly scrambled dataset was generated by scrambling values taken from the actual data—effectively eliminating potential relationships in the dataset. Average within cluster distance represents the mean distance a point is from all other points in a cluster, while average between cluster distance represent mean distance of a point from all points in another cluster. Statistically valid clustering would be shown as significant deviation of clustered point scores against a scrambled dataset where no real relationship is present. T-test: ****** $p \leq 0.0001$**

Figure S7: Representative staining of IHC markers in sub-stratified samples. Representative imaging of IHC stains taken from our 8 admixed samples to highlight variation in PD and psPD events in spite of similar overall histology categorization (control, hypercellular, and inflammatory). Samples are captured at 40x resolution from Phillips Image Management System. It can be appreciated that overall histology between events is similar, but subtle differences in staining intensity and morphology are present.

Figure S8: Volcano plot analysis of differentially expressed genes in "control" regions in PD and psPD event from GeoMx. Differential analysis of PD (right) and psPD (left) events with respect to "control" regions are highlighted in the volcano plot. Labeled genes represent those that show significant log fold change towards a direction of disease.
"Control" histology is represented as overall normal brain with typical neurons and oligodendrocytes present.

Figure S9: Immune exhaustion marker expression in CGGA and DSP datasets. Comparison of PD1 [left], TIM3 [middle], and LAG3 [right] in (A) normalized expression data taken from CGGA and (B) imputed normalized expression data taken from DSP. Gene expression of markers was imputed in the DSP samples due to missing genes. Imputation was performed using a random forest model. T-test: *** ≤ 0.05, ** ≤ 0.01, *** ≤ 0.001, **** $p \leq 0.0001$;** DSP samples were additionally evaluated for mixed effect: **PD1** (**Enhancement Status: $F(1,6) = 0.35$, $p = 0.58$, $\eta^2 = 0.05$; Histology: $F(2,179.2) = 3.35$, $p = 0.04$, $\eta^2 = 0.04$, Status-Histology: $F(2,179.2) = 14.21$, $p = 2.0e-6$, $\eta^2 = 0.14$**); **TIM3** (**Enhancement Status: $F(1,6) = 0.02$, $p = 0.89$, $\eta^2 = 0.004$; Histology: $F(2,180.6) = 15.22$, $p = 7.8e-7$, $\eta^2 = 0.14$, Status-Histology: $F(2,180.6) = 3.85$, $p = 0.02$, $\eta^2 = 0.04$**); **LAG3** (**Enhancement Status: $F(1,6) = 0.007$, $p = 0.94$, $\eta^2 = 0.001$; Histology: $F(2,178.3) = 0.29$, $p = 0.75$, $\eta^2 = 0.003$ Status-Histology: $F(2,178.3) = 1.07$, $p = 0.35$, $\eta^2 = 0.01$**).

Table S1: Clustering gene list for recurrent GB CGGA analysis.

Gene	Process	Reference Paper(s)
PROM1, FUT4, ITGA6, CD44, L1CAM, SOX2, NANOG, OLIG2, MYC, BMI1, MSI1, NES, ID1	*Stem Cells*	12-15
MKI67, POLD1, POLD2, POLD3, POLD4, PCNA, CCND1, CCNA2	*Cell Proliferation*	16-17
CD3E, GZMK, CXCR3, BCL11B, IL7R, KLRG1	*CD8 T Cells*	18
GZMA, NKG7, NCR1, SAMD3, STYK1, TBX21	*NK Cells*	18
BANK1, CD79A, CD79B, CD55, CD38, CD19	*B Cells*	18
IGFBP4, ITM2A, AMIGO2, TRAT1, CD40LG, ICOS	*CD4 T Cells*	18
NR4A3, HAVCR2, KMO, DNASE1L3, ANPEP, CXCL16	*Dendritic Cells*	18
C1QC, CD5L, FCGR3A, ITGB5, MERTK, CCL8	*Macrophages*	18
IL2RA, CTLA4, FOXP3, SLC35D1, GDPD3, CISH	*T Regs*	18
CD3D, CD3E, CD3G, CD247	*T Cell Receptor*	18
GZMA, GZMB, GZMH, GZMK, GZMM, CCL5, PRF1	*Cytotoxicity*	18
CIITA, JAK2, GBP5, IRF2, GBP4, IRF1	*Interferon Signaling*	18
HCST, IL16, IL18, CCL4, CCL3, CCL19, CCL22	*Immunoregulatory Signaling*	18

Table S2: Admixed sample lesion description and histology.

Sample	Diagnosis	Time to Enhancement (Months)	Slide Tissue Area (mm^2)	Ki67%	Side	Lobe	Lesion Size (cm)	Pathology Comments
**1**	PD	3	227.4	4	Right	Parietal	3.8x3.6	"Diffuse positive staining in cytologically atypical cells for p53 and Olig2. CD163 highlights background microglia and macrophages."
**2**	PD	1	175.0	10	Right	Parietal	4.8x4.1	"Recurrent glioblastoma with scattered very atypical mitoses noted. Surrounding brain shows evidence of treatment effect characterized by infiltrating macrophages and reactive gliosis"
**3**	psPD	1	72.1	5	Left	Frontal	3.3x2.0	"Hypercellularity is made up of CD163 positive macrophages and microglia; however, rare Olig2 positive neoplastic glioma cells are appreciated"
**4**	psPD	0	45.3	13	Left	Temporal	2.7x1.8	"Majority of cells in the tissue section are CD163 positive macrophages/microglia. However, p53 positive cells are noted with corresponding Ki67 labeling"
**5**	PD	6	171.7	25	Left	Frontal	3.0x2.0	"Fragments of hypercellular gray and white matter with abundant macrophages/microglia as well as cytologically atypical astrocytes"
**6**	PD	5	102.3	5	Right	Frontal	2.2x1.6	"Significant microglial/macrophage cells on the CD163 immunohistochemical; less than 10% of the specimen is composed of OLIG2-positive tumor cells"
**7**	psPD	0	307.1	7	Left	Temporal	4.6x3.2	"CD163 immunohistochemical stain highlights numerous infiltrating macrophages accounting for some of the hypercellularity; hypercellular fragments of residual/recurrent glioma featuring scattered atypical astrocytic cells"
**8**	psPD	6	87.5	NA	Right	Temporal	3.0x2.5	"Sections show a pleomorphic glioma with vascular proliferation, hyaline necrosis and mineralized necrosis"

Table S3: Mixed effect modeling statistics of image analysis groupings to segmentation measures.

Measured Outcome	Grouping	NumDF:DenDF	F-value	P-value	Eta-Squared
NN Distance	Status	1:5.8	0.23	0.65	0.04
	Status-Histology	2:251.6	3.66	0.03*	0.03
Ki67 Staining Ratio	Status	1:6.1	0.11	0.75	0.02
	Status-Histology	2:246.2	2.10	0.12	0.02
Olig2 Staining Ratio	Status	1:6.2	0.28	0.61	0.04
	Status-Histology	2:252.4	1.25	0.29	0.01
p53 Staining Ratio	Status	1:6	2.1	0.20	0.26
	Status-Histology	2:250.4	11.71	1.4e-5*	0.09
CD163 Staining Ratio	Status	1:277.5	215.54	2.2e-16*	0.44
	Status-Histology	2:3849.9	42.03	2.2e-16*	0.02

Table S4: Mixed effect modeling statistics of DSP analysis groupings to WGCNA and immune deconvolution measures.

Measured Outcome	Grouping	NumDF:DenDF	F-value	P-value	Eta-Squared
MEbrown	Status	1:6	0.59	0.47	0.09
	Status-Histology	2:178.5	9.83	8.95e-5*	0.1
MEpink	Status	1:6.1	1.97	0.21	0.25
	Status-Histology	2:179.8	3.92	0.02*	0.04
MEmagenta	Status	1:6	1.4	0.28	0.19
	Status-Histology	2:180.2	12.15	1.1e-5*	0.12
MEturquoise	Status	1:6	0.7	0.44	0.1
	Status-Histology	2:179.4	30.99	2.8e-12*	0.26
MEgreeenyellow	Status	1:6	1.9	0.22	0.24
	Status-Histology	2:179.4	6.31	2.2e-3*	0.07
MEred	Status	1:5.8	0.67	0.44	0.1
	Status-Histology	2:182.6	15.33	7.0e-7*	0.14
Neutrophils	Status	1:6.1	2.69	0.15	0.31
	Status-Histology	2:180.7	7.5	7.4e-4*	0.08
Naïve CD8 T-cells	Status	1:6.1	0.002	0.97	2.6e-4
	Status-Histology	2:179.7	18.18	6.5e-8*	0.17
NC Monocytes	Status	1:6.1	1.29	0.3	0.18
	Status-Histology	2:180.8	16.3	3.1e-7*	0.15
Macrophages	Status	1:6.4	3.56	0.11	0.36
	Status-Histology	2:179.1	20.49	9.8e-9*	0.19

Figure S1: Tissue processing framework for molecular studies.

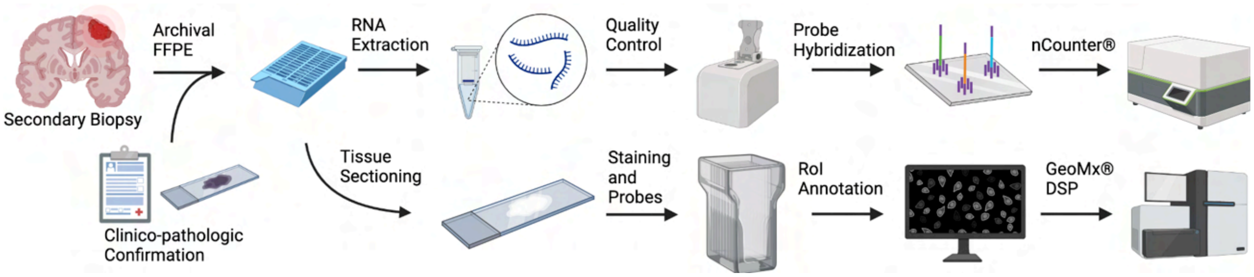

Figure S2: Top 150 DEGs from OSU PD vs psPD Cases.

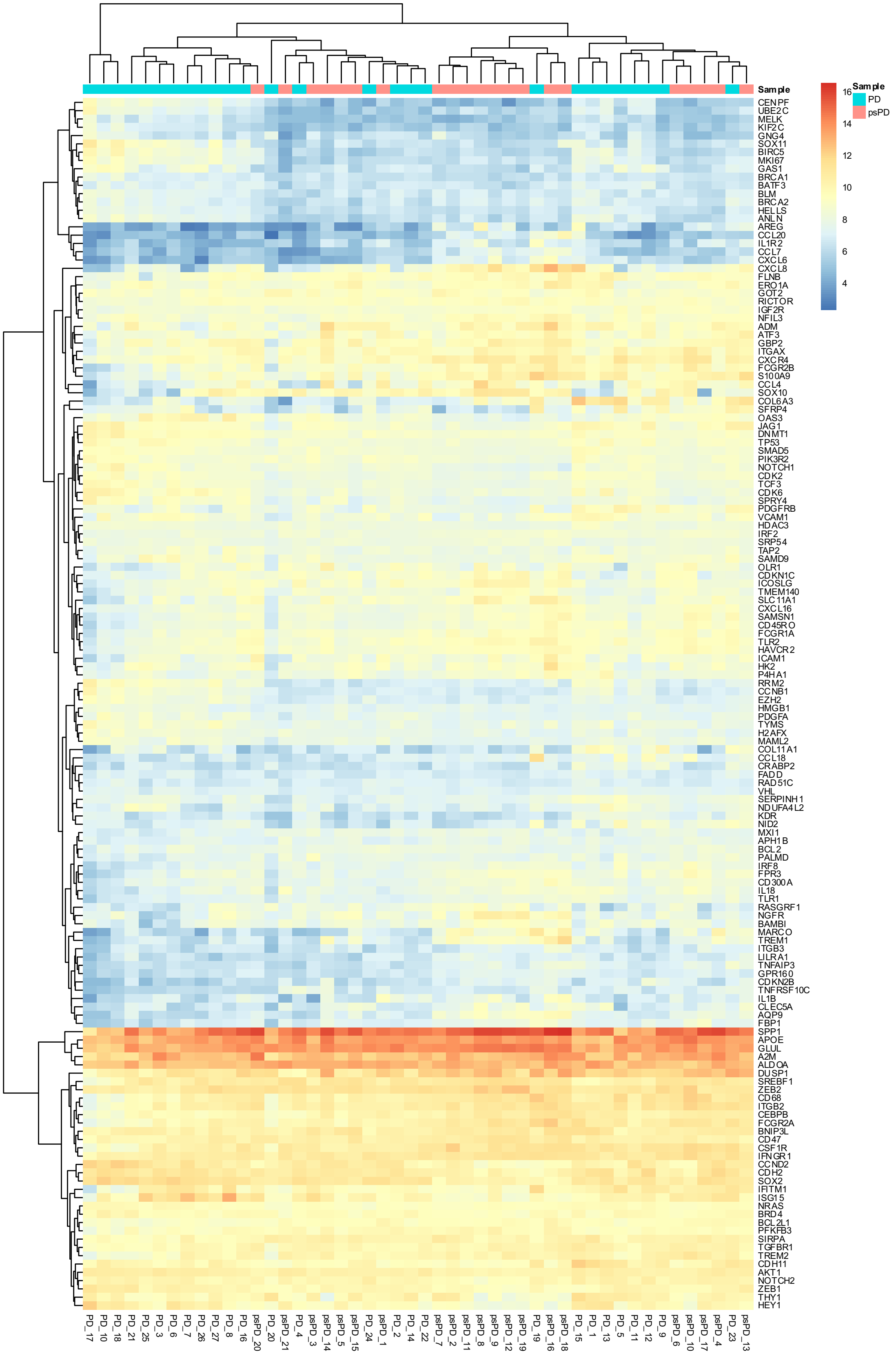

Figure S3: Image processing schematic of IHC stains.

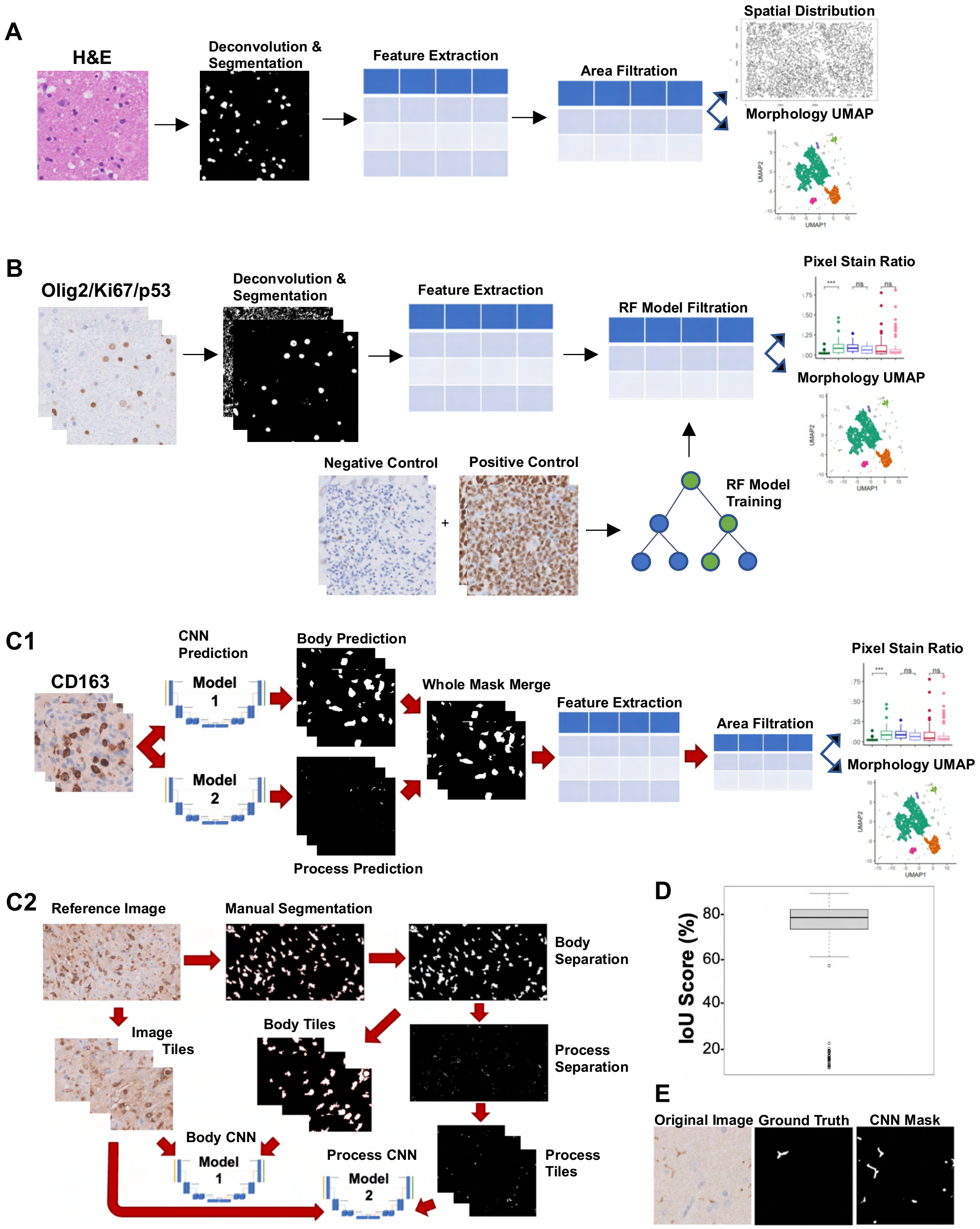

Figure S4: Cluster statistics of CGGA clustering analysis.

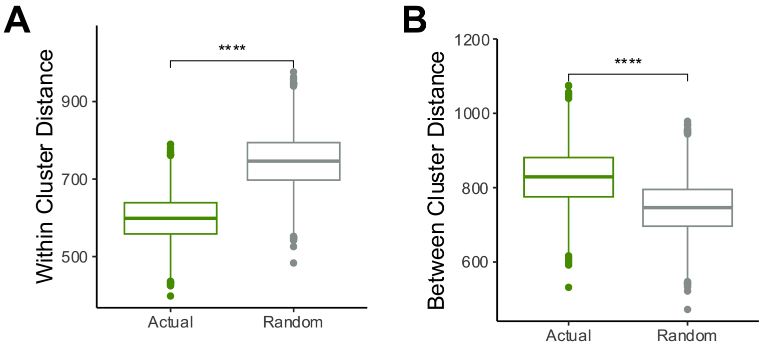

Figure S5: WGCNA module and immune proliferation gene overlap.

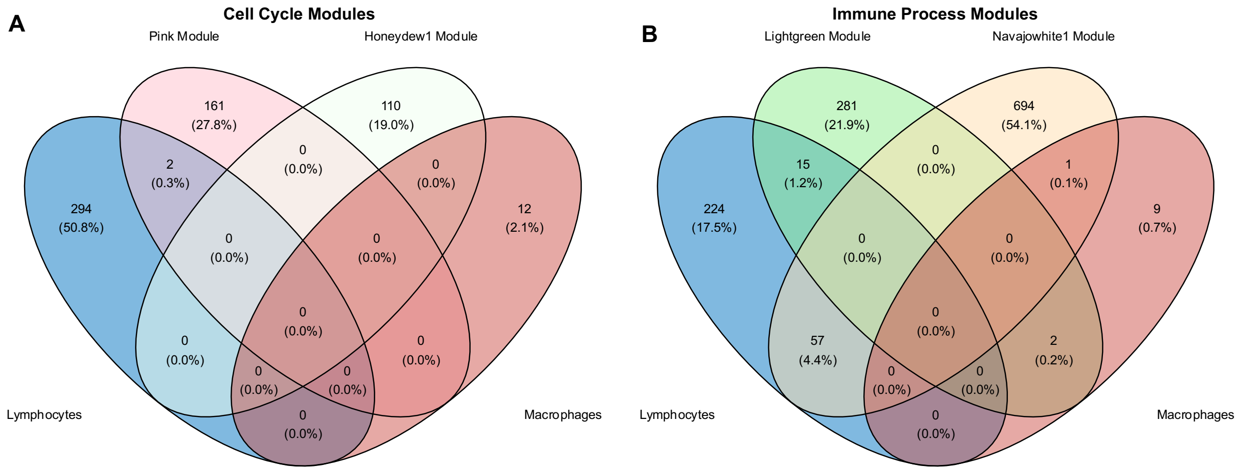

Figure S6: Cluster statistics of nCounter clustering analysis.

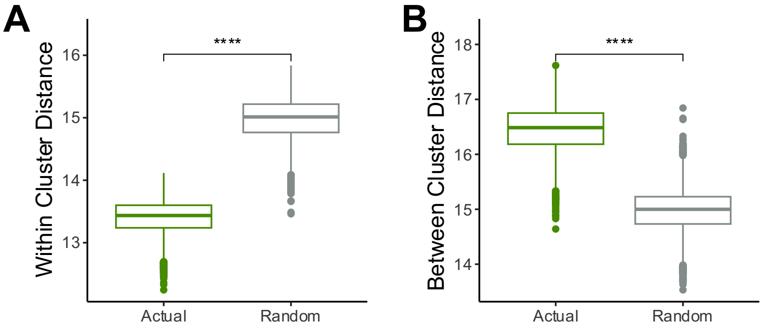

Figure S7: Representative staining of IHC markers in sub-stratified samples.

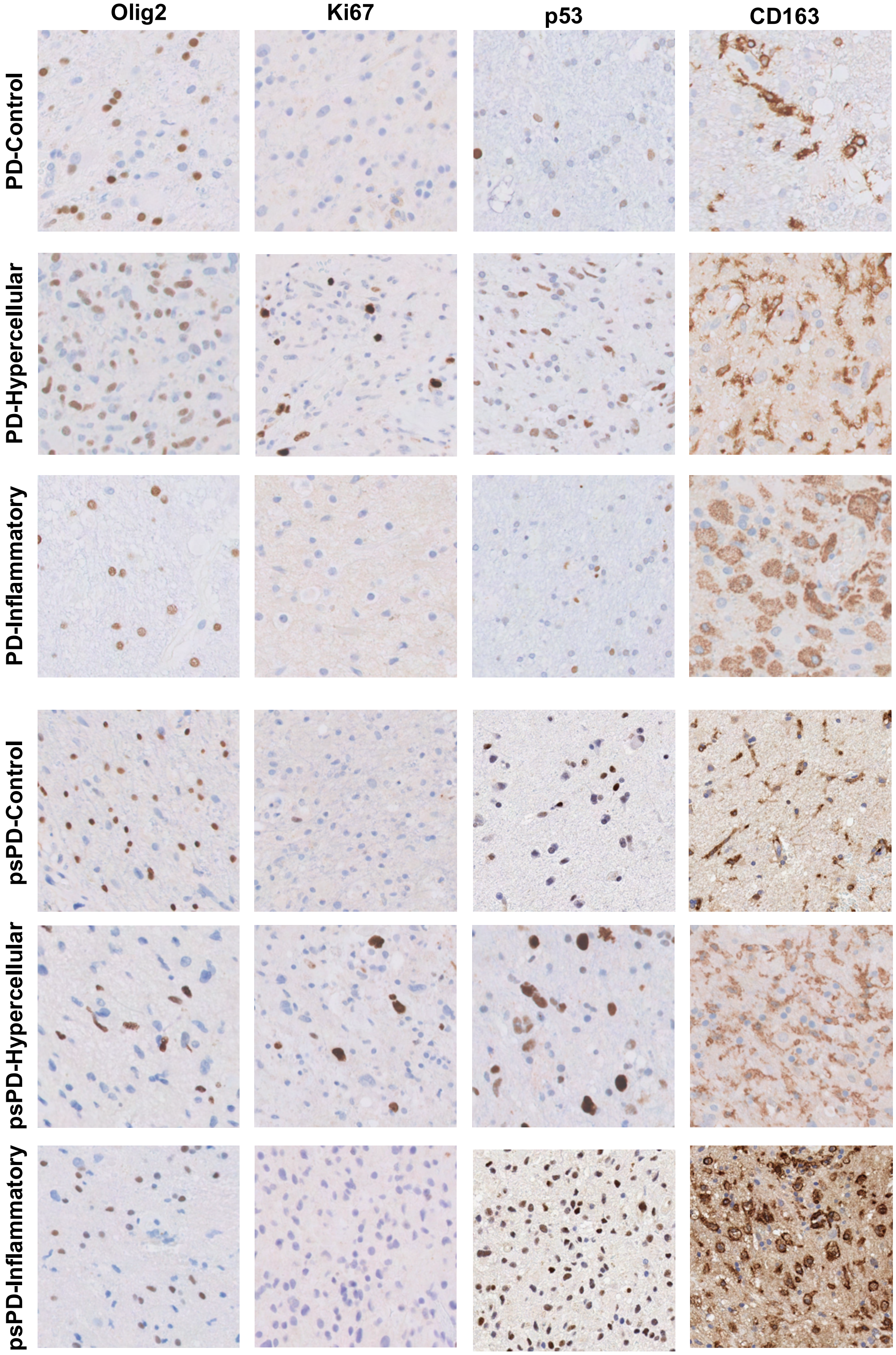

Figure S8: Volcano plot analysis of differentially expressed genes in "control" regions in PD and psPD event from GeoMx.

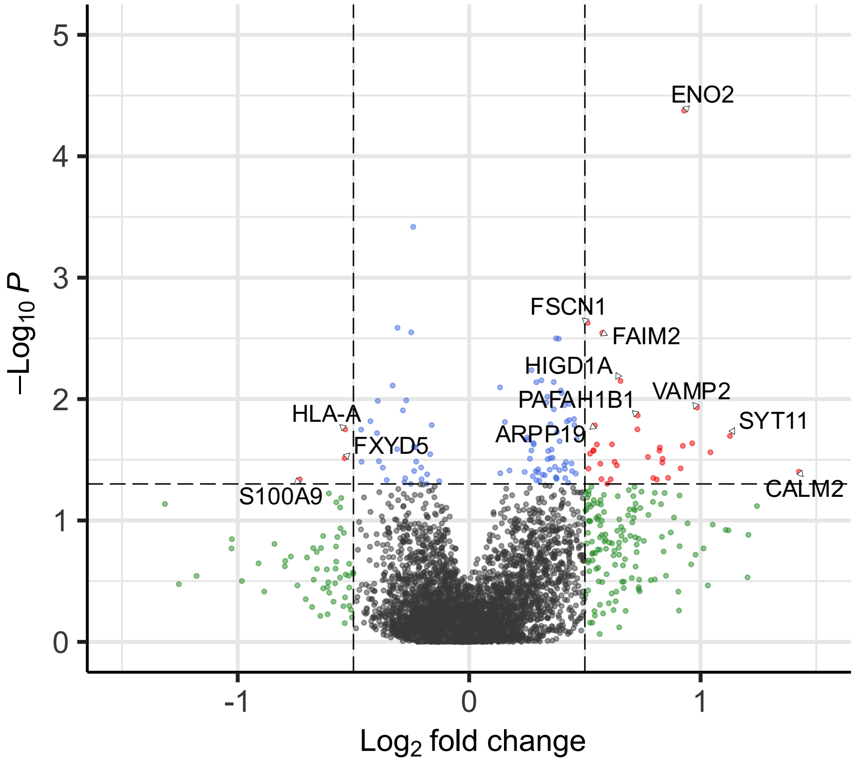

Figure S9: Immune exhaustion marker expression in CGGA and DSP datasets.

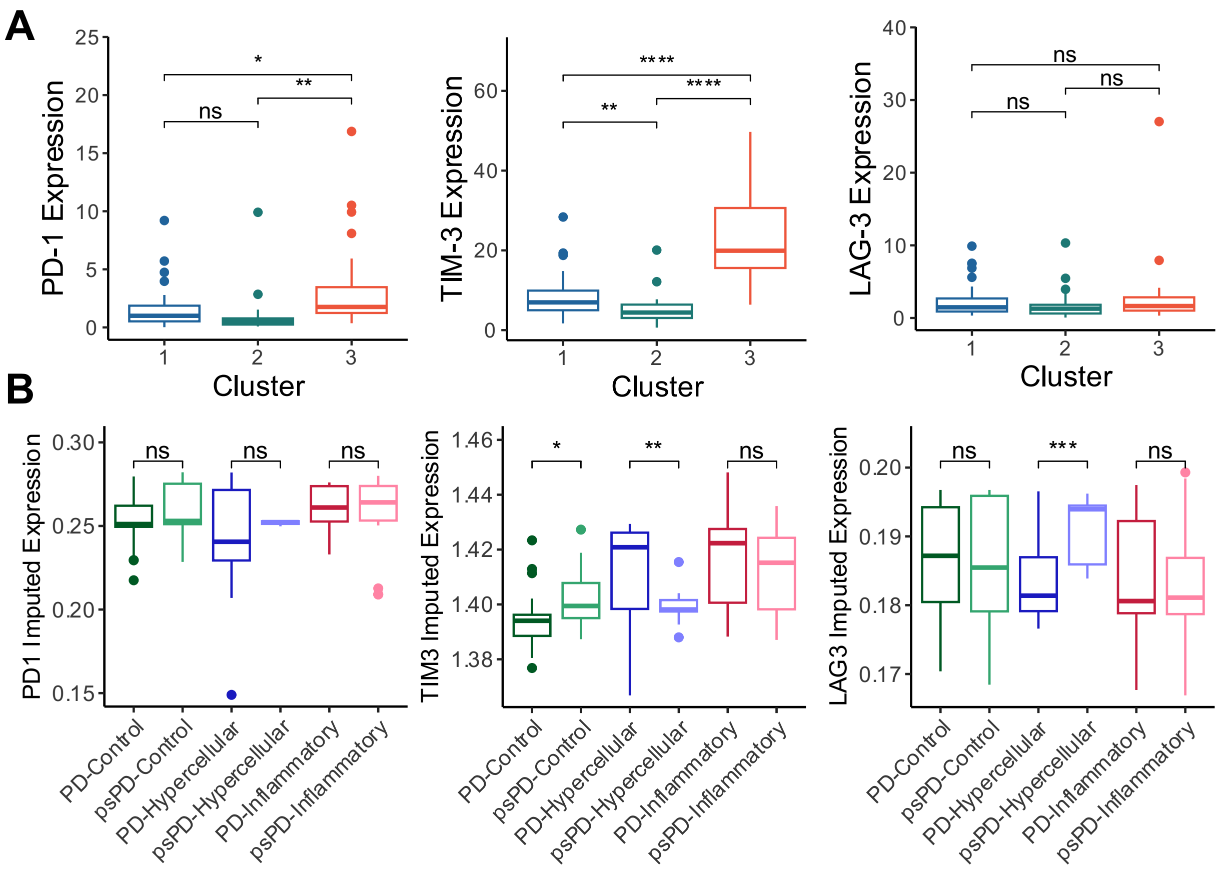

Supplement: Supplementary file 1 — Additional file 1. Table S1. Clustering gene list for recurrent GB CGGA analysis. Table S2. Admixed sample lesion description and pathology. Table S3. Mixed effect modeling statistics of image analysis groupings to segmentation measures. Table S4. Mixed effect modeling statistics of DSP analysis groupings to WGCNA and immune deconvolution measures. Figure S1. Tissue processing framework for molecular studies. Figure S2. Top 150 DEGs from OSU PD vs psPD Cases. Figure S3. Image processing schematic of IHC stains. Figure S4. Cluster statistics of CGGA clustering analysis. Figure S5. WGCNA module and immune proliferation gene overlap. Figure S6. Cluster statistics of nCounter clustering analysis. Figure S7. Representative staining of IHC markers in sub-stratified samples. Figure S8. Volcano plot analysis of differentially expressed genes in “control” regions in PD and psPD event from GeoMx. Figure S9. Immune exhaustion marker expression in CGGA and DSP datasets. [file 40478_2023_1587_MOESM1_ESM.pdf]
